# Stem microanatomical phenomic uncovers a potential role for *ZmLSM2* in regulating maize stem bending strength

**DOI:** 10.1111/jipb.70140

**Published:** 2026-01-19

**Authors:** Ying Zhang, Zejia Wang, Jianjun Du, Jiawen Li, Guanmin Huang, Yanxin Zhao, Yanru Wang, Qingmei Men, Minkun Guo, Minggang Zhang, Xianju Lu, Chuanyu Wang, Qikun Liu, Xinyu Guo, Chunjiang Zhao

**Affiliations:** ^1^ Information Technology Research Center, Beijing Academy of Agriculture and Forestry Sciences Beijing 100097 China; ^2^ National Engineering Research Center for Information Technology in Agriculture Beijing 100097 China; ^3^ Beijing Key Laboratory of Digital Plant, Beijing Academy of Agriculture and Forestry Sciences Beijing 100097 China; ^4^ Beijing Key Laboratory of Crop Molecular Design and Intelligent Breeding, Beijing Academy of Agriculture and Forestry Sciences Beijing 100097 China; ^5^ State Key Laboratory of Gene Function and Modulation Research, Beijing Advanced Center of RNA Biology (BEACON) School of Advanced Agricultural Sciences, Peking University Beijing 100871 China; ^6^ Beijing Key Laboratory of Maize DNA Fingerprinting and Molecular Breeding, Maize Research Center, Beijing Academy of Agriculture and Forestry Sciences Beijing 100097 China

**Keywords:** lodging resistance, maize, stem mechanics, vascular bundle, *ZmLSM2*

## Abstract

Modern maize stems possess a well‐developed vascular bundle system, which is critical for providing mechanical support and lodging resistance. However, characterization of the microanatomical features of vascular bundles and their functional implications in stem mechanics remains challenging, primarily due to technical limitations in high‐throughput microanatomical analysis of stem tissues. We thus constructed data sets consisting of over 500,000 maize stem CT images from a maize diversity panel of 383 inbred lines. We evaluated 32 microanatomical phenotypes of maize basal internodes across two environments in different years. By incorporating engineering mechanics parameters, we calculated novel characteristics of the vascular bundles, including the moment of area (MOA) and the polar moment of inertia (PMOI). Through the high‐density phenotypic data set, we identified multiple stem microanatomical phenotypes strongly associated with lodging resistance, particularly of vascular bundle mechanical traits. By integrating population genetic profiling, we discovered and confirmed that *ZmLSM2* (U6 small nuclear ribonucleoprotein specific Sm‐like 2) serves as a key regulator of stem mechanical strength, might function in RNA processing and maturation within vascular stem cells, identifying novel genetic targets for improving maize lodging resistance. This approach demonstrates the value of combining advanced phenotyping with multi‐omics analyses for crop improvement. These discoveries will deepen the understanding of plant stem biomechanical principles and provide novel targets for enhancing lodging resistance in crop breeding programs.

## Graphical abstract

Maize ZmLSM2 (U6 small nuclear ribonucleoprotein specific Sm‐like 2) controls maize stem strength by affecting vascular bundle structure. Using advanced computed tomography imaging and artificial intelligence identified key stem traits linked to lodging resistance and developed a predictive model. These findings offer targets for breeding stronger, higher‐yielding maize.

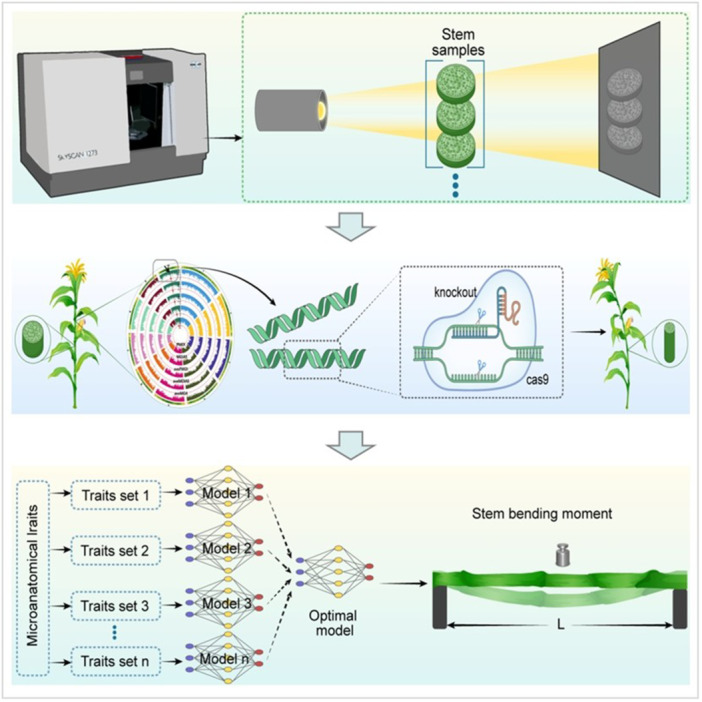

## INTRODUCTION

Maize (*Zea mays* L.), a globally important staple crop, plays a vital role in ensuring global food security (Shiferaw et al., 2011; FAO. et al., 2021; Zheng et al., 2023). Throughout domestication, maize has undergone significant trait improvements, including increased kernel number and size for higher yields, as well as development of a more compact and upright architecture to optimize photosynthesis (Doebley et al., 1997; Doebley, 2004; Wright et al., 2005; Costa et al., 2024). However, stem lodging, caused by high‐density planting and extreme weather, still poses a significant risk to maize production, with yield losses reaching up to 50% in severely affected regions (Stokstad, 2023; Zhao et al., 2024). Therefore, development of lodging‐resistant maize varieties has become a key goal in genetic improvement.

As a key lodging‐related trait, stem mechanical strength has received significant attention in maize improvement efforts (Cook et al., 2020; Stubbs et al., 2022; Huang et al., 2025). The vascular bundles, along with their associated sheath sclerenchyma, form the structural “backbone” that supports maize plants (Murdy, 1960; Lucas et al., 2013; De Rybel et al., 2016). Studies have shown that stem bending resistance is closely linked to anatomical features, including cortex thickness, total vascular bundle count, and individual vascular bundle area (Niklas, 1998; Huang et al., 2016; Stubbs et al., 2022; Li et al., 2024b). However, due to challenges in the accurate quantification of stem microanatomical traits at scale, the precise influence of these features on stem mechanical strength remains unclear (Huang et al., 2016; Zhao and Zhou, 2021).

Traditional mechanical tests, such as rind penetration resistance (RPR) and three‐point bending, provide direct measurements of the mechanical properties and lodging resistance of maize stem (Shah et al., 2017). However, lodging resistance is a complex trait influenced by multiple stem biological and biophysical properties, including stem diameter, vascular bundle number and distribution, sclerenchyma fiber density, and cell wall composition (Wang et al., 2020; Muszynska et al., 2021; Zhao et al., 2024). These biological features are likely governed by distinct genetic pathways. For example, *Stiff1* was shown to regulate cellulose and lignin deposition in the rind region and vascular bundles, thereby affecting stem strength (Zhang et al., 2020). Consequently, individual gene effects may only weakly correlate with the overall stem mechanical strength (Muszynska et al., 2021). To fully elucidate the genetic regulation of stem biomechanical properties, it is essential to deploy advanced microanatomical trait quantification methods and systematically analyze these biological components.

Several methods for the detection of stem anatomical structures have been developed, including microscopic photography, computed tomography (CT) scanning, and magnetic resonance imaging (Manga‐Robles et al., 2021; Wu et al., 2021; Zhang et al., 2021; Li et al., 2024b). Our previous work established a micro‐CT‐based phenotyping method that enables non‐destructive, three‐dimensional visualization of various internal stem anatomical traits (Zhang et al., 2021). When combined with sophisticated imaging analysis tools, CT technology shows potential for use not only for the analysis of how individual stem anatomical structures influence overall mechanical strength but also for facilitating trait mapping to improve maize lodging resistance through genetic approaches.

In this study, we quantified over 32 stem microanatomical traits through CT scanning in a maize diversity panel consisting of 383 inbred lines and revealed the relationships between these microanatomical traits and stem mechanical strength. We performed a GWAS that identified *ZmLSM2* (Zm00001eb416130) as a key regulator of maize vascular bundle development and lodging resistance. The epigenetic feature associated with *ZmLSM2* gene expression was also investigated. Furthermore, we developed a prediction model for maize stem mechanical strength using machine learning algorithms, achieving a predictive correlation coefficient greater than 0.7. These findings demonstrate how advanced phenotyping technologies combined with population genomic analyses can effectively dissect complex crop traits and identify key genetic regulators for crop improvement. The high‐density stem CT image data sets presented here offer a powerful resource for several pivotal applications: Artificial intelligence‐driven agricultural image analytics, statistical genomics mining, and predictive modeling of carbon–water–nutrient allocation dynamics.

## RESULTS

### Characterization of stem microanatomical traits in a maize diversity panel

**Figure 1:**
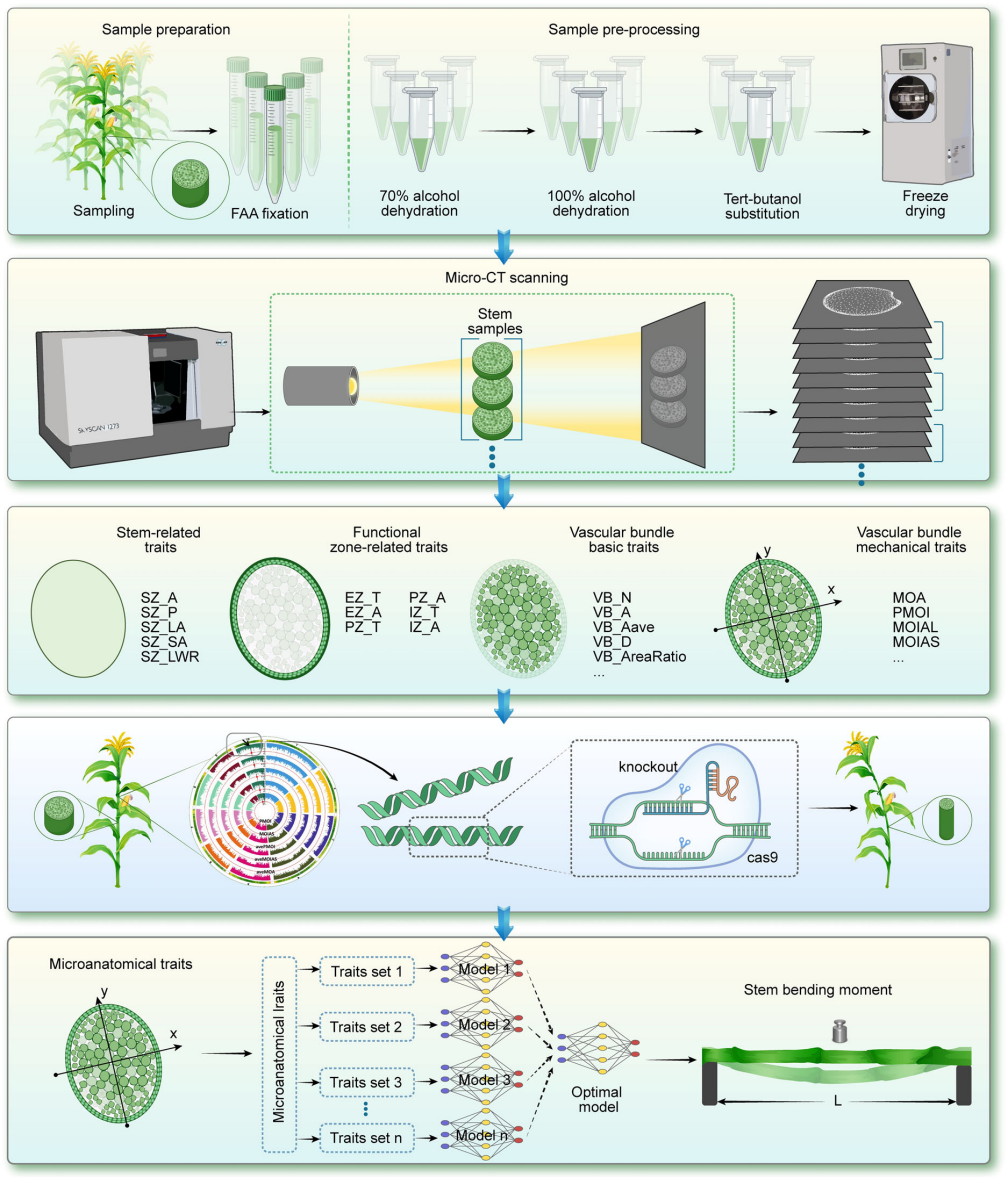
Standardized experimental pipeline integrating sample preparation, stem anatomical phenotyping, automated image processing, data extraction, GWAS, and gene functional validation The first line represents sample collection and sample pre‐processing. The second line represents the high‐throughput microscopic images obtained by CT scanning of stem material. The third line represents that 32 microanatomical traits that were systematically quantified using our in‐house automated analysis platform. The fourth line represents the GWAS analysis and functional verification of candidate genes. The last line represents the prediction model construction for maize stem bending strength using varying numbers of microanatomical traits.

To investigate the biological basis of maize stem bending strength, we developed a standardized experimental pipeline integrating sample preparation, stem anatomical phenotyping, automated image processing, data extraction, GWAS, and gene functional validation (Figure 1). We modified a previously developed micro‐CT imaging protocol to enable rapid, high‐resolution data acquisition and standardized processing across CT platforms (see Materials and Methods; Zhang et al., 2021). Using this system, we captured cross‐sectional images of the third, panicle, and apex internodes from 383 maize inbred lines grown in Beijing and Sanya during two growing seasons (year 2018 and 2021); more than 100 sequence CT images were obtained for each sample, generating over 500,000 CT images with 15 μm resolution (Tables S1, S2).

For the single CT image, we systematically quantified 32 microanatomical traits using our in‐house automated analysis platform, obtaining multiscale parameters of stem cross‐sectional architecture (Figure 1; Table S3). In addition to basic morphological indicators, such as vascular bundle area, number, and density, we also incorporated geometric parameters, including the moment of area (MOA), the area moment of inertia (MOIA), and the polar moment of inertia (PMOI). We hypothesized that these geometric parameters better characterize the dimensions and spatial distribution of vascular bundles, thereby providing a more accurate representation of maize stem mechanical properties. We have conducted a data set‐wide validation focusing on vascular bundle number and size‐related traits of the stem slice. Vascular bundle number identification achieved *R* ^*2*^ = 0.9916 and size‐related traits measurements achieved *R* ^*2*^ > 0.97. These results demonstrate the reliability of our pipeline for core phenotypic extraction (Figure S1). We evaluated 32 microanatomical phenotypes of base internodes and estimated best linear unbiased prediction (BLUP) values across both environments to represent each line's stem and vascular bundle phenotypes (Tables S4, S5).

### Clustering and geographical distribution of maize stem microanatomical traits

**Figure 2:**
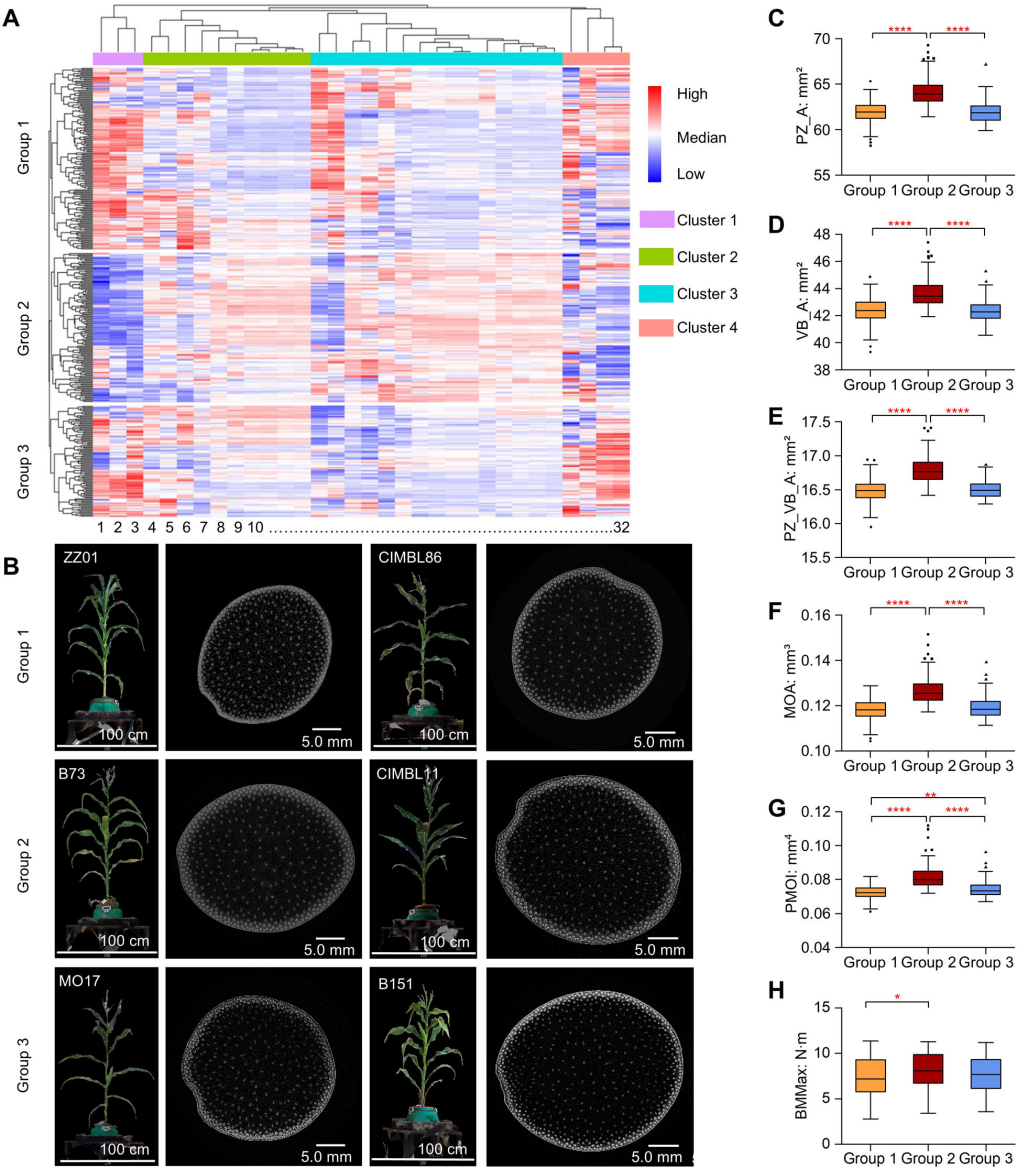
Changes in vascular bundles phenotypes of stems among the association mapping panel composed of 383 diverse inbred lines **(A)** Cluster analysis of vascular bundle traits of base stems in the maize association mapping panel. 32 traits were grouped into four categories (column); the first category (purple bar) includes IZ_VB_D, VB_D, and PZ_VB_D (coding for 1–3), the second category (green bar) includes VB_N, PZ_VB_N, IZ_VB_N, IZ_VB_A, SZ_LA, SZ_SA, IZ_A, IZ_T, SZ_A, and SZ_P (coding for 4–13), the third category (blue bar) includes VB_AreaRatio, IZ_VB_AreaRatio, PZ_A, PZ_T, VB_Aave, PZ_VB_A, aveMOIAS, avePMOI, aveMOA, aveMOIAL, VB_A, MOIAS, MOIAL, PMOI, and MOA (coding for 14–28), and the fourth category (red bar) includes four indicators SZ_LWR, PZ_VB_AreaRatio, EZ_A, and EZ_T (coding for 29–32). 383 inbred lines can be classified into three subgroups through clustering based on the 32 phenotypic traits (row), Group 1 represented by ZZ01 and CIMBL86, Group 2 represented by B73 and CIMBL11, and Group 3 represented by MO17 and B151. **(B)** Plant three‐dimensional point cloud images and CT images of the third internodes of ZZ01, CIMBL86, B73, CIMBL11, MO17, and B151. Box plot for **(C)** PZ_A, **(D)** VB_A, **(E)** PZ_VB_A, **(F)** MOA, **(G)** PMOI, and **(H)** BMMax of three groups for 383 inbred lines. Intergroup comparisons were performed using ordinary one‐way ANOVA of multiple comparisons; *P < 0.05 and ***P < 0.0001.

To elucidate interrelationships among maize stem microanatomical traits, we performed cluster analysis on all 32 traits, which grouped them into four distinct categories based on their correlations (Figure 2A). Category 1 comprised vascular bundle density traits, including PZ_VB_D, VB_D, and IZ_VB_D (abbreviation and description in Table S3). Category 2 encompassed traits related to vascular bundle number, spatial distribution, and cross‐sectional dimensions such as IZ_VB_N, IZ_VB_A, SZ_SA, SZ_LA, IZ_A, IZ_T, SZ_A, and SZ_P. Category 3 had both structural and mechanical traits, including mechanical properties (MOA, PMOI, MOIAL, MOIAS, avePMOI, aveMOA, aveMOIAL, and aveMOIAS), vascular bundle area metrics (VB_AreaRatio, IZ_VB_AreaRatio, VB_Aave, and PZ_VB_A, VB_A), and periphery zone dimensions (PZ_A and PZ_T). Category 4 included epidermis zone‐ and shape‐related traits such as EZ_A, EZ_T, SZ_LWR, and PZ_VB_AreaRatio. Notably, mechanical traits from Category 3 showed strong correlations with vascular bundle area metrics and periphery zone dimensions (Figure 2A), indicating their potential importance in determining stem mechanical strength.

Population cluster analysis partitioned the 383 inbred lines into three phenotypically distinct groups (Figure 2A, B; Tables S4, S5). Group 1 (represented by ZZ01 and CIMBL86) displayed thin stems with narrow periphery zones, sparse vascular bundles, reduced vascular bundle area, and low mechanical inertia (PMOI and MOA) (Figures 2A, B, S1). In contrast, Group 2 (represented by B73 and CIMBL11) showed robust stems with broad periphery zones, dense vascular bundles, large vascular bundle area, and high mechanical inertia (Figures 2A, B, S1). Group 3 (represented by MO17 and B151) showed intermediate mechanical and anatomical traits (Figures 2A, B, S2). Quantitatively, Group 2 outperformed Group 1 by 3.6%–12.4% across different microanatomical traits (Figures 2C–G, S2). Three‐point bending tests confirmed these trends, with Group 2 showing the highest average maximum bending moment (BMMax, 8.107 N·m), followed by Group 3 (7.845 N·m) and Group 1 (7.459 N·m) (Figure 2H).

The maize diversity panel comprised maize temperate germplasms from China and the United States as well as tropical/subtropical germplasms (TST) from CIMMYT (Table S1). CIMMYT lines showed significantly greater stem cross‐sectional dimensions (SZ_A), vascular bundle area (VB_A), periphery zone size (PZ_A), and total vascular bundle counts (VB_N), but lower vascular bundle density (VB_D) compared to Temperate germplasm, suggesting a trade‐off between vascular bundle number and spatial distribution (Table S6). Consistent with these anatomical advantages, CIMMYT lines dominated Group 2 (Figure S3), highlighting the genetic potential of TST germplasm for improving lodging resistance in maize breeding programs.

### Microanatomical Determinants of Stem Bending Strength

To identify the microanatomical traits most strongly associated with maize stem bending strength, we quantified the mechanical properties of the third internodes, including the bending moment at maximum load (BMMax) and the bending moment at 10 mm displacement (BM10 mm). Parallel quantification of 32 microanatomical traits revealed significant heterogeneity in their correlations with bending strength, ranging from weak to very strong (*R* ^*2*^ = 0.003 to 0.649) across all traits (Figures S4, S5).

**Figure 3:**
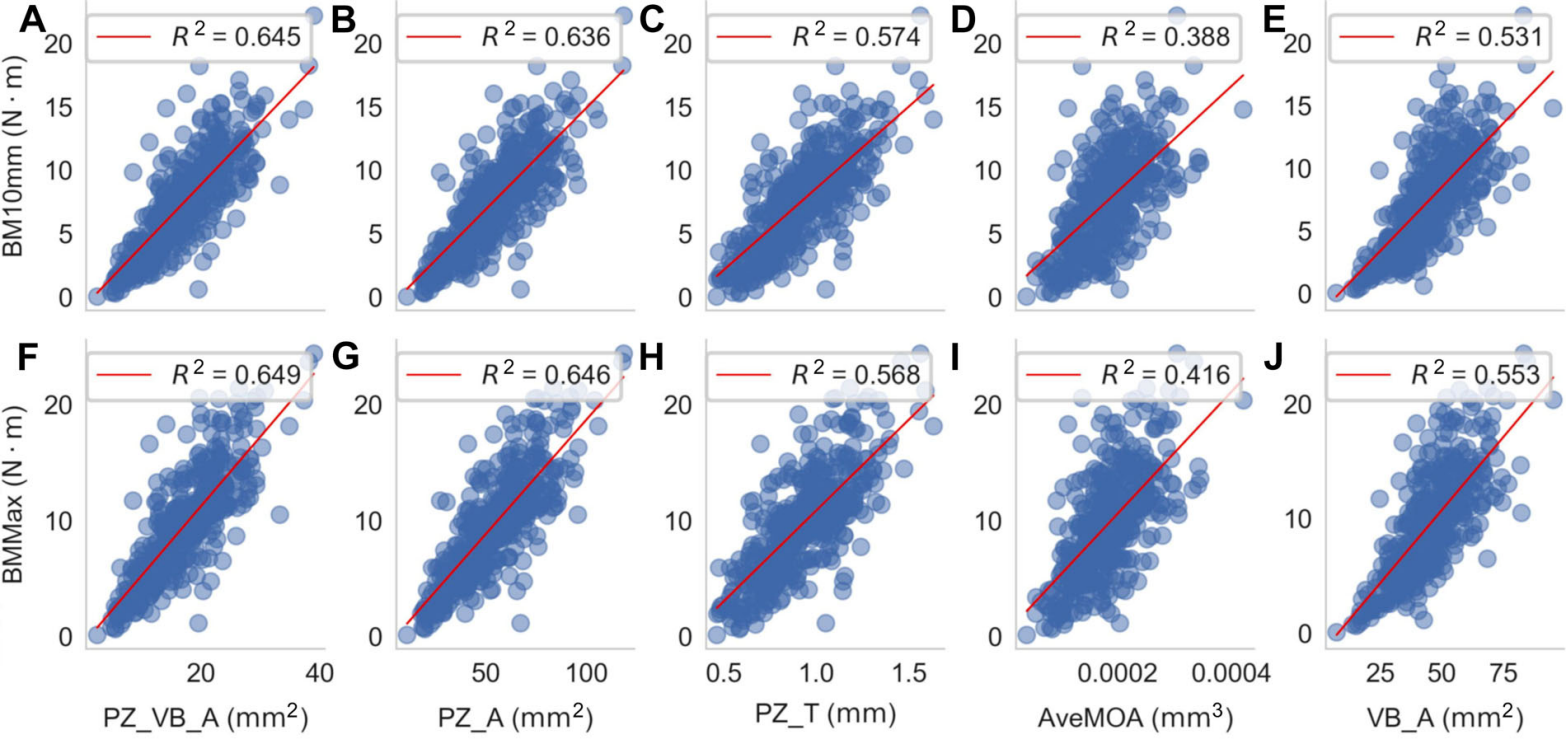
The plot shows linear regression values between five microanatomical traits with BM10 mm and BMMax **(A)** PZ_VB_A, **(B)** PZ_A, **(C)** PZ_T, **(D)** MOA, and **(E)** VB_A vs. BM10 mm. Each dot represents a sample measurement. The red line indicates the linear regression fit. **(F)** PZ_VB_A, **(G)** PZ_A, **(H)** PZ_T, **(I)** MOA, and **(J)** VB_A vs. BMMax. Each dot represents a sample measurement. The red line indicates the linear regression fit.

The recursive feature elimination (RFE) algorithm prioritized five traits with the highest correlations: Total vascular bundle area (VB_A), vascular bundle area in the peripheral zone (PZ_VB_A), peripheral zone thickness (PZ_T), peripheral zone area (PZ_A), and vascular bundle MOA (Figures 3, S6, and S7). These results indicate that microanatomical features, such as greater vascular bundle area, particularly in the peripheral zone, are strongly correlated with enhanced stem bending strength.

By enabling high‐throughput, precise, and fully automated phenotyping of stem microanatomical traits, this pipeline dissects maize stem mechanical strength, a complex agronomic trait, into biologically interpretable components. These quantifiable components now provide a foundation for systematic genetic dissection.

### Identification of microanatomical trait‐related genes

**Figure 4:**
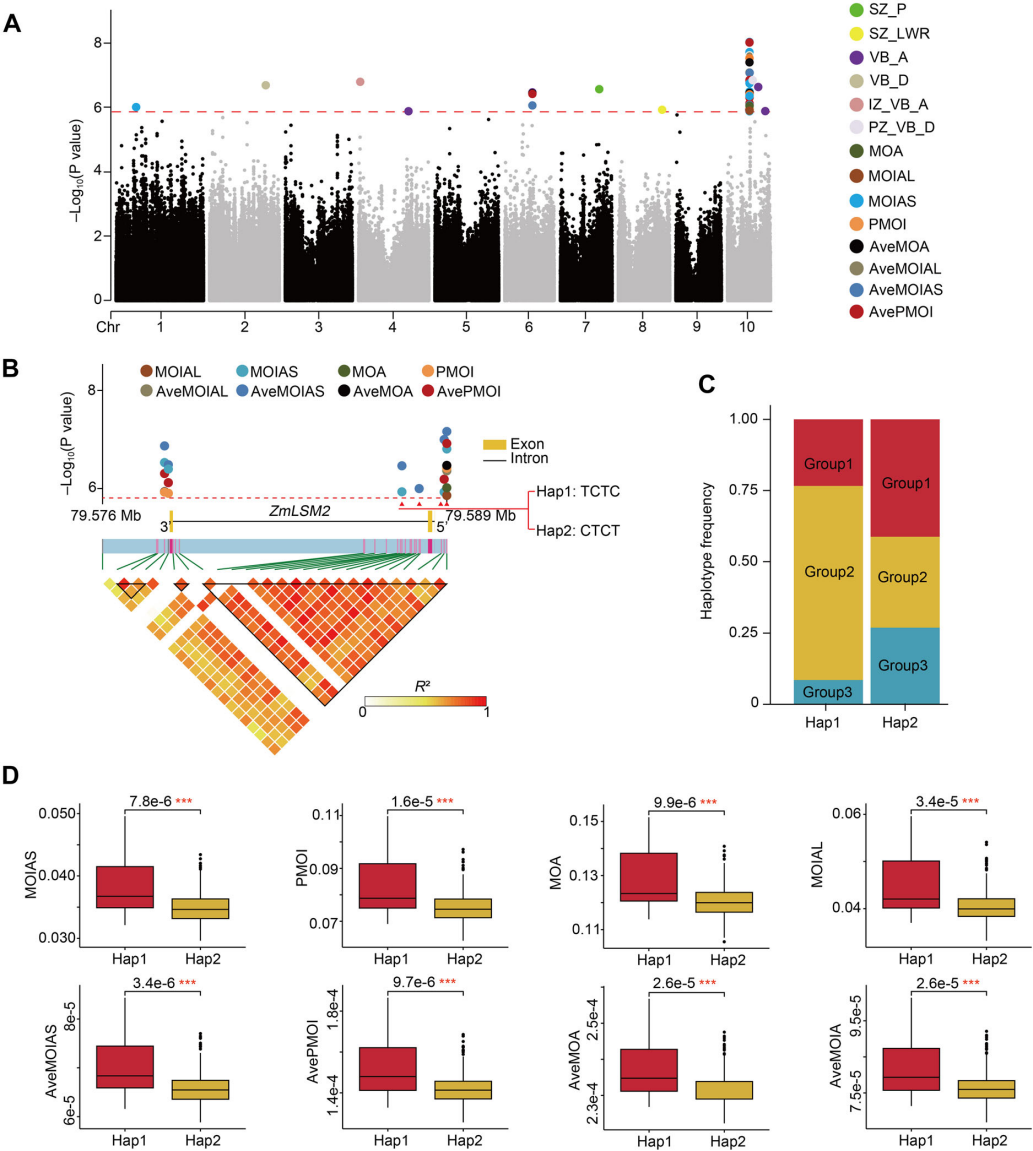
GWAS identification of candidate genes for variation in maize stem and vascular bundle traits **(A)** Manhattan plot of 14 stem and vascular bundle traits analyzed by GWAS. **(B)** Local Manhattan plot (top) and LD heatmap (bottom) surrounding *ZmLSM2* on chromosome 10. Red triangles indicate the SNP significantly associated with the trait. Black‐outlined triangles mark the strong linkage intervals. **(C)** Frequency distributions of the four significantly associated SNPs of *ZmLSM2*. The 383 inbred lines were classified into three subgroups based on the 32 phenotypic traits. Group 1 is represented by ZZ01 and CIMBL86, Group 2 is represented by B73 and CIMBL11, and Group 3 is represented by MO17 and B151. This classification is consistent with Figure 2. **(D)** Box plot for MOIAS, PMOI, MOA, MOIAL, aveMOIAS, avePMOI, aveMOA, and aveMOIAL of two haplotypes of *ZmLSM2*. Intergroup comparisons were performed using the two‐sided Mann–Whitney–Wilcoxon test, with statistical significance (*P* < 0.001) denoted by triple asterisks.

Here, the heritability of the 32 phenotypic traits of maize stem and vascular bundles based on the data from two environments was calculated. The 32 phenotypic traits showed different heritability patterns, ranging from 0.455 to 0.721 (Table S7). To identify the genetic basis underlying maize stem microanatomical traits, we conducted genome‐wide association analyses (GWAS) for each of the 32 traits using GEMMA (Genome‐wide Efficient Mixed Model Association algorithm; significance threshold: *P* < 1.5446 × 10^−6^). We identified 18 significant single‐nucleotide polymorphisms (SNPs) associated with 14 traits (Figure 4A; Table S8). By prioritizing candidate genes within 50 kb upstream and downstream of these SNPs, we revealed 14 GWAS candidate genes (Table S8; see the Materials and Methods). These genes explained 6.58%–9.14% of phenotypic variance (median: 7.34%) and were enriched in pathways related to vascular bundle morphogenesis, cellulose synthesis, cell elongation/differentiation, and stimulus responses (Table S8). Notably, we detected known vascular developmental genes, such as *ABI3‐VP1* (Zm00001eb421100), which showed a significant association with the total vascular bundle area (VB_A) (Table S8). This gene encodes a B3 domain protein that regulates cell elongation, cellulose synthesis, and vascular differentiation (Swaminathan et al., 2008; Zhang et al., 2021). Another key candidate, *ZmLSM2* (*LSM‐like 2*, Zm00001eb416130), was associated with multiple mechanical traits (PMOI, MOIA, and MOA) through six SNPs (Figure 4A; Table S8) and was selected for further study.

*ZmLSM2* encodes a small 93‐amino acid protein within a 9,998‐bp gene comprising three exons, and two exons separated by a 9,716 bp intron (Figure 4B). In Arabidopsis, LSM family proteins are predominantly expressed in vascular tissues and form heteroheptameric complexes involved in mRNA decay and pre‐mRNA splicing (Perea‐Resa et al., 2012). *Arabidopsis lsm* mutants show multiple developmental defects, including disorganized vein formation and reduced lateral root development, suggesting a conserved role in vascular development. Among the six SNPs, four (chr10.s_79589399, chr10.s_79587706, chr10.s_79589300, and chr10.s_79588357) are significantly linked to *ZmLSM2* and cluster in the gene 5′ region defining two haplotypes: hap1 (TCTC) and hap2 (CTCT) (Figure 4B). Hap1 was predominant in Group 2 accessions, which typically develop stems with enhanced mechanical strength (Figures 2C–H, 4C, D). In contrast, hap2 accessions showed reduced stem strength across all measured traits (PMOI, MOIAS, MOIAL, MOA, etc.; Figure 4D). Given the strong haplotype–mechanical trait associations and *ZmLSM2*'s putative role in vascular development, we propose *ZmLSM2* as a key genetic regulator of maize stem mechanical strength.

### Functional verification of *ZmLSM2* in regulating vascular bundle mechanical strength

**Figure 5:**
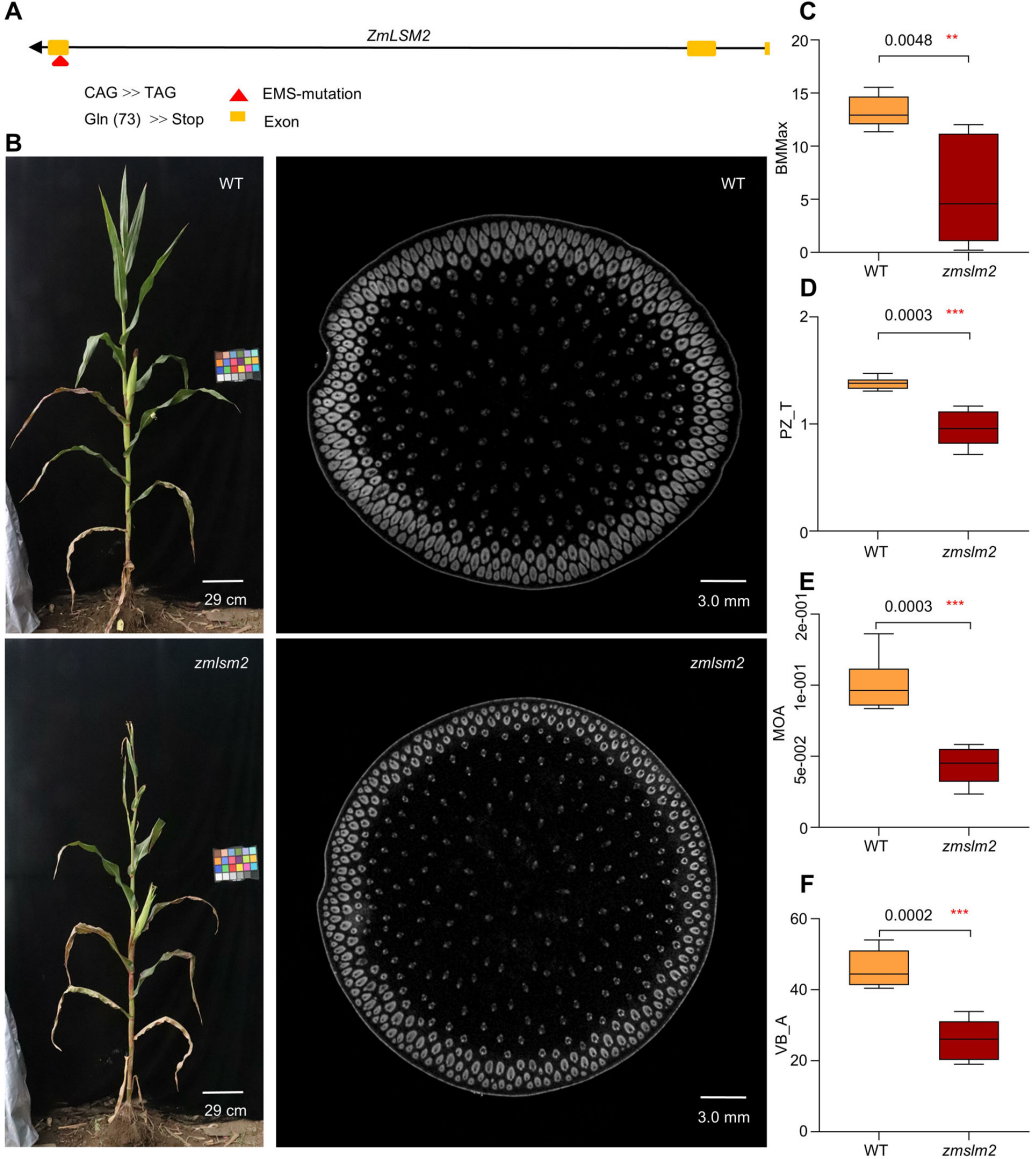
**The** ***zmlsm2*** **EMS mutation caused the microanatomical trait changes of stem and vascular bundles and stem mechanical properties in maize** **(A)** *ZmLSM2* gene structure and position of the EMS mutation (the red box marks the mutation site). **(B)** Field‐grown WT (wild type) and *zmlsm2* mutant plants (the scale bar corresponds to 29 cm), and the CT scanning images of base internodes from WT and *zmlsm2* mutant plants at the silking stage (the scale bar corresponds to 3 mm). **(C)** BMMax **(D)** PZ_T **(E)** MOA, and **(F)** VB_A of the base internodes from *zmlsm2* mutant and WT maize plants at the silking stage (*n* = 10). Phenotyping of the two groups were compared using Student's *t*‐test (*P < 0.05 and **P < 0.01).

To confirm *ZmLSM2*'s role in regulating maize vascular bundle microanatomical structure and stem mechanical properties, we obtained a *zmlsm2* EMS mutant in the B73 background (Mut_Sample EMS5‐0ab9e0), which carries a C‐to‐T substitution at nucleotide 3,312 position, introducing a premature stop codon at amino acid 74 position (Figure 5A). The decrease in *ZmLSM2* expression was detected by quantitative reverse‐transcription PCR (qRT‐PCR) in the *zmlsm2* mutant plants (Figure S8D). The *zmlsm2* mutant showed reduced plant height and thinner stems compared to wild‐type B73 controls (Figures 5B, S8A–C, E, F). At the silking stage, the average plant height of the wild types was 230.3 cm, while that of the *zmlsm2* mutants was 200.4 cm. The average stem diameter of wild types at the third internodes was 19.91 mm, while that of the *zmlsm2* mutants was 15.73 mm. Three‐point bending tests demonstrated significantly lower stem bending moments in the mutants than in wild‐type plants (Figures 5C, S9). Additionally, *zmlsm2* mutants showed thinner peripheral zones (PZ_T), smaller vascular bundle areas (VB_A), and reduced MOA of vascular bundles (Figures 5D–F, S8, and S9).

**Figure 6:**
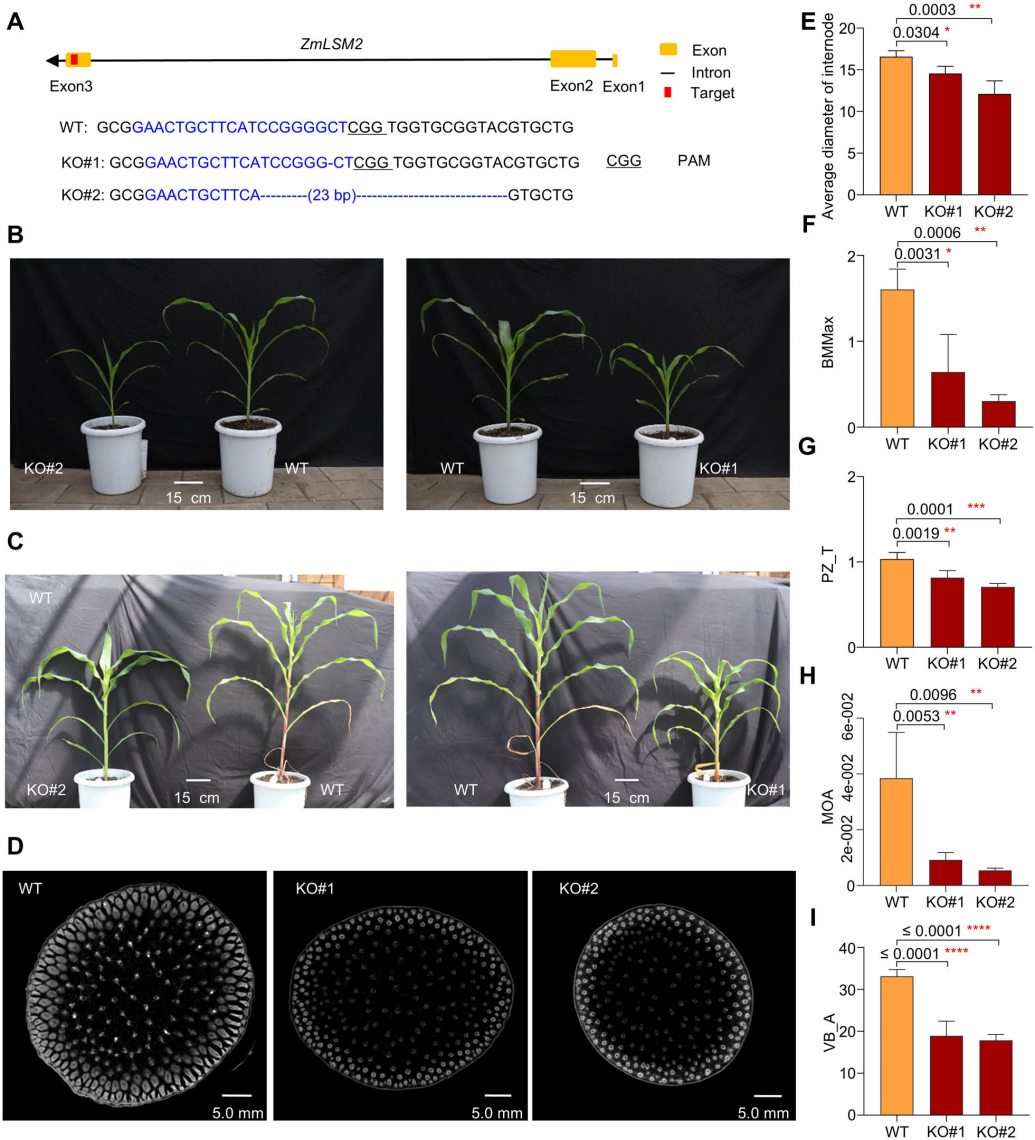
The *zmlsm2* knockout mutants caused microanatomical trait changes in stem and vascular bundles and stem mechanical properties in maize **(A)** Sequences of two homozygous knockout lines with deletions in target sites that truncated the *zmlsm2* ORF (KO#1 and KO#2). The wild‐type sequence is shown at the Top. Target sites and PAM sequences are highlighted in blue and underline, respectively, and deletions are indicated by dashes. The sequence gap length is shown in parentheses. **(B)** Growth of knockout lines and wild‐type plants in the greenhouse at the V9 stage. **(C)** Growth of knockout lines and wild‐type plants in the greenhouse at the V12 stage. **(D)** CT scanning images of base internodes from knockout lines and wild‐type plants at the V12 stage (the scale bar corresponds to 5.0 mm). **(E)** Average diameter of internode, **(F)** BMMax, **(G)** PZ_T, **(H)** MOA, and **(I)** VB_A of the base internodes from knockout lines and wild‐type plants at the V12 stage (WT, *n* = 10, KO#1, *n* = 8, KO#2, *n* = 10). Phenotyping of the two groups was compared using Student's *t*‐test (*P < 0.05, **P < 0.01, and ****P < 0.0001).

We also generated a *zmlsm2* knockout mutant in the B73 background using CRISPR/Cas9 technology, obtaining two independent knockout lines (KO#1 and KO#2) (Figure 6A–C). Similar to the *zmlsm2* EMS mutants, these CRISPR mutant lines showed significantly thinner stems (Figures 6B–E, S10) and lower bending moments compared to their corresponding non‐transgenic control plants (Figure 6F); also, a decrease in *ZmLSM2* expression was detected by qRT‐PCR in *zmlsm2* knockout mutant plants (Figure S11). Both vascular bundle area and MOA, as well as peripheral zone thickness (PZ_T), were reduced in the knockout lines at both the 12th leaf stage (Figure 6H–I) and the maturity stage (Figure S12). Together, these results confirm *ZmLSM2*'s role in regulating stem microanatomical traits and mechanical strength in maize.

### Contrasting epigenetic features of *ZmLSM2* in maize germplasms

Our findings establish *ZmLSM2* as a critical regulator of stem mechanical properties in maize. Intriguingly, while B73 is a typical group 2 germplasm that shows superior stem mechanical properties, it carries the hap2 SNP genotype typically associated with reduced stem strength (Figure 4B, D). This suggests that these SNPs alone are insufficient to determine maize stem mechanical traits. To further investigate potential regulatory mechanisms of *ZmLSM2*, we conducted detailed characterization of *ZmLSM2* using germplasms representing different stem microanatomic trait groups: B73 (group 2), ZZ01 (group 1), and CIMBL86 (group 1) (Figures S13, S14, and S15).

**Figure 7:**
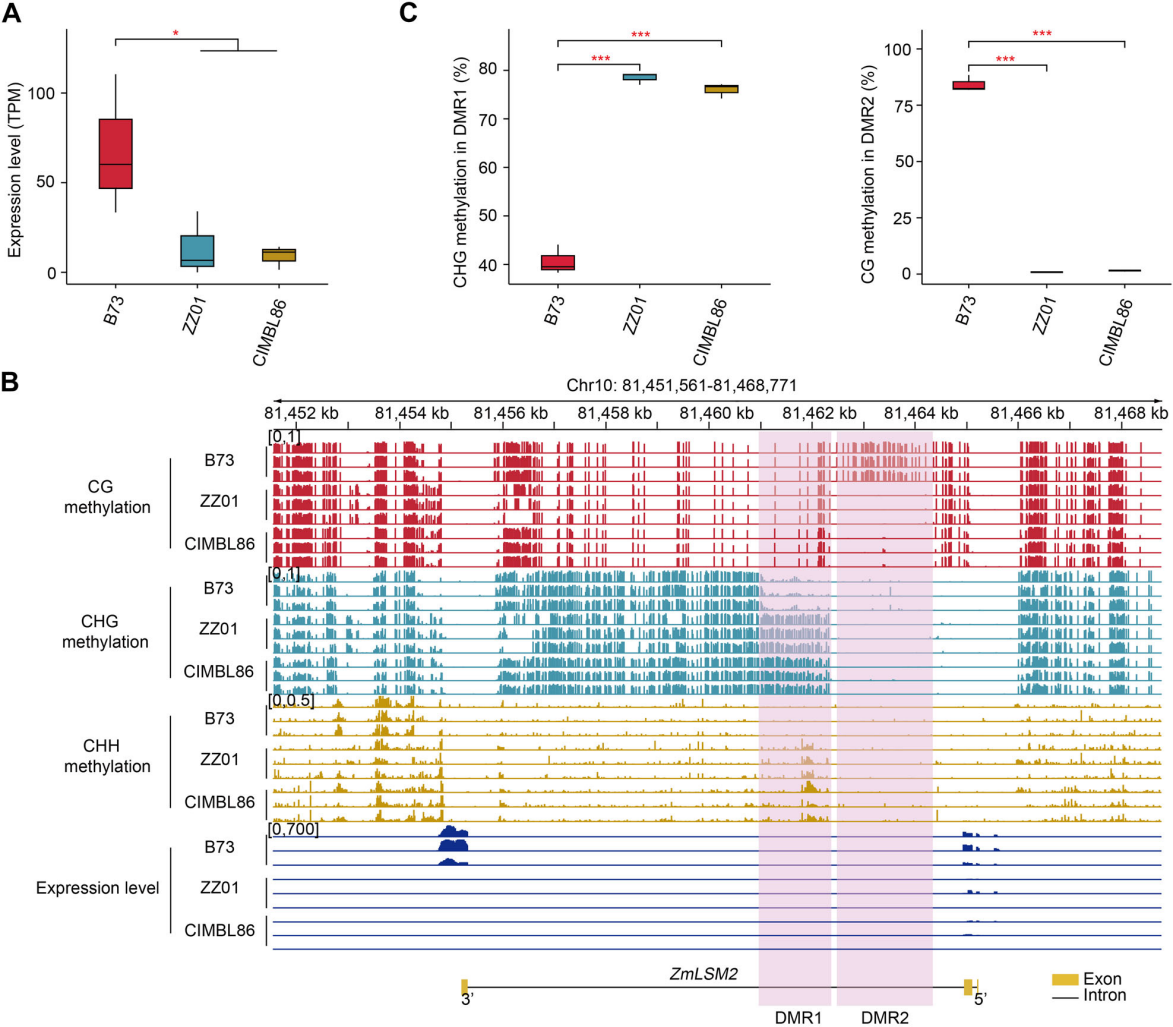
DNA methylation and gene expression of three representative cultivars **(A)** Boxplots showing the expression level of *ZmLSM2* in B73, ZZ01, and CIMBL86. Red asterisks represent statistically significant differences between the groups under comparison (Mann–Whitney–Wilcoxon test, *P < 0.05). The boxes of the boxplots indicate the upper and lower quartiles. The middle line and the individual points of the boxplots represent the median and outliers, respectively. **(B)** Genome browser view showing the levels of CG (red track), CHG (light blue track), and CHH (orange track) methylation and gene expression (dark blue track). The pink shading highlights the differentially methylated regions (DMRs). **(C)** Same as in **(A)**, except for the methylation levels in DMR1‐2 (***P < 0.001).

Notably, sequence analysis revealed complete identity in the *ZmLSM2* coding region and flanking sequences (1 kb upstream/downstream) among all three accessions, indicating an absence of alternative SNPs despite their shared hap2 genotype (Table S9). However, transcriptome analysis demonstrated significantly higher *ZmLSM2* expression in B73 compared to ZZ01 and CIMBL86 (Figure 7A), suggesting that reduced expression may contribute to their weaker stem strength. This transcriptional divergence in the absence of coding sequence variation implies possible differential regulation by trans‐acting factors or epigenetic mechanisms.

DNA methylation, a well‐characterized epigenetic modification regulating gene and TE transcription, contributes to phenotypic plasticity in plants (Kawakatsu et al., 2016; Xu et al., 2019; Cao et al., 2023; Guo et al., 2023). Our whole‐genome bisulfite sequencing (WGBS) of stem tissues from these germplasms showed high reproducibility, with PCA clearly separating genotypes while maintaining tight intra‐group clustering (Figure S16). Localized methylation analysis identified two differentially methylated regions (DMRs) in *ZmLSM2*'s intron that distinguish B73 from ZZ01/CIMBL86 (Figure 7B). Compared to ZZ01 and CIMBL86, B73 showed lower levels of CHG and CHH methylation in DMR1 but higher levels of CG methylation in DMR2 (Figure 7B, C). These contrasting methylation patterns suggest an epigenetic regulatory mechanism for stem strength, though causal relationships require further validation.

### A prediction model for maize stem bending strength

**Figure 8:**
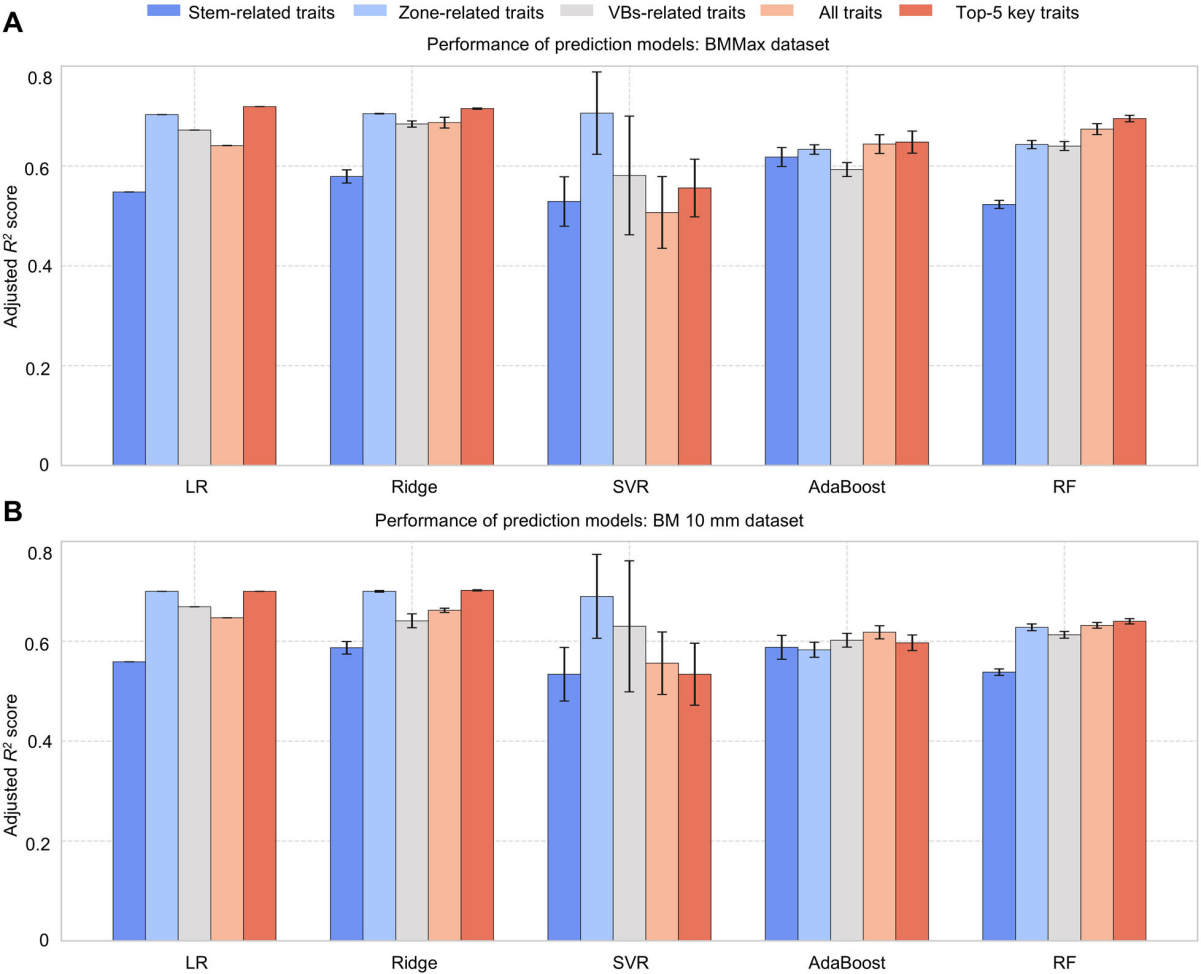
Machine learning‐based regression modeling for predicting the bending movement of stems **(A)** Machine learning‐based regression modeling for predicting the bending moment based on the BMMax of stem. **(B)** Machine learning‐based regression modeling for predicting the bending moment base on the BM10 mm of stem. The machine learning‐based regression models include AdaBoost, LR, RF, Ridge, and SVR. Feature sets for training and modeling include: (i) stem‐related traits: SZ_A, SZ_P, SZ_LA, SZ_SA, and SZ_LWR; (ii) zone‐related traits: EZ_A, EZ_T, PZ_A, PZ_T, IZ_A, and IZ_T; and (iii) VBs‐related traits: vascular bundle‐basic traits and vascular bundle‐mechanical traits. (iv) All traits were all 32 micro phenotypic indicators (See as Table S3). (v) Top five key traits: VB_A, PZ_VB_A, PZ_T, PZ_A, and MOA.

Our study demonstrates that maize stem microanatomical features are strongly associated with stem mechanical properties. To quantify this relationship, we developed predictive models using varying numbers of microanatomical traits. We evaluated five machine learning regression approaches, including linear (Ridge regression) and nonlinear methods (Random Forest, AdaBoost, SVR, and Neural Networks). Among these, Ridge regression showed the highest predictive accuracy and the lowest RMSE (Figure 8A, B; Table S10), outperforming nonlinear models. This performance pattern indicates a primarily linear relationship between stem microanatomical features and bending strength.

Remarkably, use of just the top five most predictive microanatomical traits achieved prediction precision values of 0.72 for BMMax and 0.70 for BM10 mm. Importantly, incorporation of additional traits did not further improve precision (Figure 8A, B). Compared to conventional macroscopic morphological indicators (e.g., stem cross‐section traits), these five key microanatomical features enhanced prediction accuracy by 15% (*P* = 0.0013, Figure 8A, B). These results establish peripheral zone characteristics and vascular bundle architecture as the most informative microanatomical predictors of maize stem bending strength. These robust biomarkers enable more accurate analysis of complex stem mechanical properties than traditional morphological approaches.

## DISCUSSION

Recent advances in imaging technologies have revolutionized the study of plant development and plant–environmental interactions (Zhang et al., 2024b). In this study, we leveraged micro‐CT imaging to establish a high‐throughput high‐resolution platform for quantification of maize stem microanatomical traits. The superiority of micro‐CT lies in its ability to rapidly generate three‐dimensional reconstructions of internal stem architecture with minimal sample preparation, while simultaneously capturing both morphological and mechanical parameters (Wu et al., 2021; Du et al., 2022). The automated workflow demonstrated here, including image acquisition and data processing, overcomes the limitations of traditional optical microscopy and manual sectioning methods while maintaining high precision (Table S11). Such high‐throughput capability enables population‐scale screening of lodging‐resistant germplasm, a critical need for breeding climate‐resilient crops (Hu et al., 2023). In this study, we generated a high‐resolution (15 μm) CT image data set comprising over 500,000 scans of the third, panicle, and apex internodes. Our data set represents the most extensive collection of maize inbred line stem images to date, both in terms of sample diversity and data volume (Piovesan et al., 2021; Zhang et al., 2024b). These data enable modeling and simulation of key physiological processes, including water/nutrient transport and carbon deposition. This resource provides unprecedented opportunities to bridge cellular‐scale anatomical features with whole‐plant physiological functions, advancing our understanding of plant adaptation to environmental change.

Vascular bundles serve dual functions: facilitating the transport of nutrients and water while providing mechanical support to maintain stem erectness. In monocots such as maize, vascular bundles show distinct spatial organization: Those in the central region are larger and primarily dedicated to water transport, whereas the smaller, more densely distributed peripheral bundles play a critical role in stem rigidity (Lucas et al., 2013). During maize domestication, selective pressures likely optimized vascular bundle arrangements to balance efficient water conduction with lodging resistance (Murdy, 1960). Traditional metrics focusing solely on isolated geometric features, such as vascular bundle counts and area, fail to capture the synergistic effects of spatial organization on stem rigidity (Li et al., 2024a). This resource facilitates the development of next‐generation digital plant laboratories, where in silico biology and computational modeling may substantially accelerate research cycles while reducing experimental expenditures.

To better characterize the developmental and distributional features of vascular bundles associated with lodging resistance, we incorporated several microanatomical traits based on torque‐related metrics, including PMOI, MOA, MOIA, etc. These geometric indicators are hypothesized to address a fundamental gap in current phenotyping systems by quantifying how vascular bundle quantity and spatial arrangement collectively contribute to mechanical reinforcement, thereby more accurately reflecting stem bending resistance. In this study, candidate genes identified by GWAS mainly encode enzymes involved in cell wall metabolism, transcription factors, protein kinase, and proteins related to plant signal transduction and stress response. Notably, based on the GWAS results, torque‐related traits were overrepresented among the microanatomical traits linked to *ZmLSM2*, further underscoring their functional significance (Figure 4A; Table S8).

The *LSM2* gene belongs to the snRNP Sm protein family, encoding a protein that binds to the 3′‐terminal U‐tract of U6 snRNA, participating in pre‐mRNA splicing, a process essential for RNA metabolism and gene expression regulation. The *lsm1a lsm1b* double mutant in Arabidopsis shows pleiotropic developmental defects, including delayed seed germination, abnormal vein formation, and reduced secondary root formation (Perea‐Resa et al., 2012). Although stem rigidity was not measured in these mutants, the wide spectrum of observed developmental abnormalities suggests that *Arabidopsis LSM* genes may also affect vascular development. Two lines of experimental evidence support this hypothesis: First, *Arabidopsis LSM8* shows expression in both leaf and root vascular tissues (Perea‐Resa et al., 2012); second, the reduced number of secondary roots in *lsm1a lsm1b* mutants indicates defects in pericycle cell function, which are known to be responsible for lateral root development (Péret et al., 2009). Therefore, we propose that in both eudicots and monocots, *LSM* genes likely play a conserved role in regulating stem anatomical development and mechanical properties through their function in RNA processing and maturation within vascular stem cells.

Beyond genetic variation, accumulating evidence indicates that heritable epigenetic modifications also contribute to phenotypic variation in crops (Zhang et al., 2024a; Xue et al., 2025). DNA methylation plays a critical role in maintaining genome integrity through transcriptional silencing of transposable elements. Additionally, studies have demonstrated that DNA methylation influences pre‐mRNA splicing (Lev Maor et al., 2015). In our analysis of *ZmLSM2*, we identified a large, heavily methylated intron between exon 2 and exon 3 (Figure 7B). Notably, two differentially methylated sites were identified within this intron. In plant genomes, CG methylation is commonly found within transcribed gene regions, a phenomenon known as “gene body methylation” (GBM). Generally, plant GBM is not associated with gene silencing. Instead, it is often linked to constitutively or moderately expressed genes and is thought to potentially stabilize gene expression or prevent spurious transcription (Ou et al., 2019) On the other hand, non‐CG DNA methylation (CHG and CHH) is often deposited *de novo* through the RNA‐directed DNA methylation (RdDM) pathway and is associated with the silencing of TEs and genes (Muyle et al., 2022; Luo et al., 2025). The established functional distinction between CG methylation, which is non‐repressive in the context of gene bodies, and repressive non‐CG methylation aligns with the differential accumulation of these marks at the *ZmLSM2* locus. We propose that the high *ZmLSM2* expression in B73 is permitted by low CHG/CHH methylation in DMR1. This feature, rather than the variation in CG methylation at DMR2, appears to be the key regulatory determinant.

Alignment of our WGBS reads at the *ZmLSM2* locus did not reveal abnormal patterns, such as large gaps, insertions, or a high frequency of split reads. To further validate this, we cloned and Sanger‐sequenced the entire *ZmLSM2* genomic region from both B73 and ZZ01. This sequencing confirmed the absence of any SVs. We detected only one SNP, located at the 3′ end, 2,529‐bp downstream of the *ZmLSM2* stop codon. Next, we investigated the presence of transposable elements (TEs) in multiple maize germplasm using published reference genomes, including that of B73, CHANG7‐2, SK, YE478, Mo17, and CML228. *De novo* TE prediction at the *ZmLSM2* locus using EDTA software (Zhang et al., 2018) did not detect any positive signal, suggesting that the region is likely free of detectable transposons. Interestingly, upon examining our RNA‐seq data, we found that *ZmLSM2* transcripts possibly undergo alternative transcriptional terminal in different maize germplasms, as shown in Figure S17. Specifically, in B73, where *ZmLSM2* is highly expressed, the transcripts extend across the very large intron to encompass the terminal exon (exon 3). In contrast, in ZZ01 and CIMBL86, where *ZmLSM2* expression is low, the transcripts are only present at the 5′ end of the gene, suggesting early transcriptional termination. It is possible that the early termination in ZZ01 and CIMBL86 is caused by the enrichment of non‐CG DNA methylation in these lines. In summary, our additional analyses suggest that major SVs or TEs are unlikely to be the underlying cause of the observed differences. Instead, these findings raise the possibility that DNA methylation‐dependent alternative splicing could underlie functional variation in *ZmLSM2* across maize germplasms.

This work underscores the transformative potential of combining advanced imaging technologies with genomic resources to decode the genetic basis of complex agronomic traits. By bridging the resolution gap between macroscopic field observations and molecular genetic analyses, our approach provides a vital tool for accelerating the development of lodging‐resistant crop varieties. Moreover, the stem CT image data sets presented here provide an unprecedented platform for three pivotal applications: (i) Artificial intelligence‐driven agricultural image analytics; (ii) statistical genomics mining of mechanical support determinants; and (iii) predictive modeling of carbon–water–nutrient allocation dynamics. These resources collectively advance targeted identification of genetic architecture underlying complex agronomic traits, offering a prioritized target for future plant adaptation to environmental change design.

## MATERIALS AND METHODS

### Population materials and growth conditions

A maize (*Zea mays* L.) association panel of 383 genetically diverse inbred lines was prepared, including tropical, subtropical, and temperate materials representing global maize diversity (Yang et al., 2010). These inbred lines can be divided into four subgroups (SS, NSS, TST, and mixed) according to their genetic relationship, and three subgroups (China, CIMMYTI, and USA) according to the geographical origin (Li et al., 2013; Table S1). The 383 maize inbred lines were grown under field conditions in 2018 at Tongzhou Experimental Station of Beijing Academy of Agriculture and Forestry Science in Beijing (116°28′E, 39°69′N) and in 2021 at Sanya Nanfan Experimental Base of Beijing Academy of Agriculture and Forestry Science in Hainan Province (109°19′E, 18°39′N). In each environment, the experiment was conducted using a completely randomized block design with three replications. Each inbred line was planted in a four‐row plot; the distance between rows was 60 cm and the inter‐plant distance was 25 cm. The third internodes, panicle internodes, and apex internodes of three plants for each inbred line were collected at the silking period (73 d after sowing) for phenotypic traits research. The data sets based on 383 inbred lines (obtained in Beijing and Sanya, 2018 and 2021, respectively) were used for genome‐wide association analysis.

Maize hybrids (84 varieties) and inbred lines (63 inbred lines) from different areas were planted in the experimental field of the Beijing Academy of Agriculture and Forestry Sciences in 2024 (Table S12). Each variety or inbred line was planted in a two‐row plot; the distance between rows was 60 cm and the inter‐plant distance was 25 cm. The third internodes of plants were collected at the silking period (73 d after sowing) for the micro‐CT imaging and three‐point bending test. The data sets of the third basal internodes (> 400 stem samples) were used to build mechanical prediction models.

### Phenotyping of stem vascular bundle based on micro‐CT

A standardized procedure of maize microdata acquisition was developed to ensure the reliability and consistency of image acquisition.

1. Dehydration. FAA fixed stem samples were dehydrated in 70% ethanol and 100% ethanol successively for 24 h each, and then subjected to dehydration substitution with tert‐Butanol. Specifically, the samples were soaked in 100% tert‐Butanol for 24 h for the first time, and after replacing the 100% tert‐Butanol with fresh solution, they were soaked again for another 24 h.

2. Ultra‐low‐temperature drying. After completion of tertbutyl alcohol replacement, samples were removed and placed in a −80°C refrigerator and frozen for 24 h. Then, frozen samples were taken out and placed in the sample compartment of an ultra‐low‐temperature freeze‐drying instrument (LGJ‐10E, China) and freeze‐dried at −80°C for 2 h.

3. CT scanning. Dried stem samples were scanned by Skyscan 1172 and Skyscan 1273; the unified scanning parameter was set as 40 kV/250 μA and the imaging pixel size was set as 15 μm. The scanning time was 22 min per three samples for Skyscan 1172 and 12 min per five samples for Skyscan 1273. Raw image data reconstructed using Skyscan NRecon software (Bruker, Nazareth, Belgium) and more than 100 sequence CT images (8‐bit image files, BMP) were obtained for each sample.

### Image segmentation and trait extraction

The phenotyping pipeline was used to extract rich and diverse traits of stem internodes from CT images. The pipeline consisted of three steps: detection of vascular bundles, identification of zones, and phenotyping (Du et al., 2022). In the given CT image, the maize stem could be divided into three zones: the epidermis (EZ), periphery (PZ), and inner (IZ) zones. The phenotyping of vascular bundles was divided into a two‐stage task. In the first stage, all candidate regions of vascular bundles were extracted using the semantic segmentation model and used to identify vascular bundles. Each candidate region was identified mainly in the second stage according to its geometric and morphological features. An adaptive watershed‐based approach was used to identify these candidate regions.

Based on an analysis of vascular bundle morphology and geometric properties, we innovatively introduced engineering mechanics parameters, including vascular bundle MOA and vascular bundle polar moment of inertia (PMOI).

(1) $$\text{MOA }=\;\Sigma \left(\text{Ai}\times {\text{yi}}^{2}\right)$$

(2) $$\text{PMOI }=\;\Sigma \left(\text{Ai}\times {\text{ri}}^{2}\right)$$

where Ai is the area of a single vascular bundle, yi is the distance from the centroid of the vascular bundle to the neutral axis, and ri is the distance from the centroid of the vascular bundle to the centroid of the cross‐section.

### Stem three‐point bending measurements

Three‐point bending tests for maize internodes were carried out using an Instron universal testing machine (Model 3343, Norwood, Massachusetts, USA) with a 2,000 N load cell (2519‐104 Series, Norwood, Massachusetts, USA). Instron software (Bluehill, 3.0) was used to control the load cell and managed data acquisition. Stem specimens were placed between two supports, and the loading anvil was displaced at a rate of 20 mm/min. The support span of stem samples was 40–50 cm, at least 10× the sample diameter (Shah et al., 2017).

Mechanical properties of the stem, including the elastic modulus, the maximum bending load, the bending load at a bending displacement of 10 mm, can be obtained directly. The bending moment is calculated based on the bending load, and the formula of bending moment is as follows:

(3) BMMax=F(max)×L/4

where *F*(max) is the maximum bending load and *L* is the support span of stem samples.

(4) BM10mm=F(bendingloadatbendingdisplacementof10mm)×L/4

where *F* (bending load at a bending displacement of 10 mm) is the bending load at a bending displacement of 10 mm and *L* is the support span of stem samples.

### Prediction of stem bending strength using VB traits based on machine learning

The recursive feature elimination (RFE) algorithm prioritized five traits with the highest correlations from 32 phenotypic traits. We used RFE with fivefold cross‐validation. Hyperparameters (e.g., number of trees = 100 and depth = 10 for Random Forest) were optimized via grid search based on prior benchmarks and minimal RMSE. For example, tree depth was limited to prevent overfitting, and the number of trees ensured stability. The models achieved *R* ^*2*^ > 0.9, indicating reliability. We divided stem micro‐phenotypic indicators into five categories for training and modeling: (i) Combined traits were all 32 phenotypic indicators; (ii) stem‐related traits included five traits SZ_A, SZ_P, SZ_LA, SZ_SA, and SZ_LWR; (iii) VBs‐related traits included Vascular bundle (VB)‐basic traits and Vascular bundle (VB)‐mechanical traits, a total of 21 traits; (iv) zone‐related traits included six traits: EZ_A, EZ_T, PZ_A, PZ_T, IZ_A, and IZ_T; and (v) five top key phenotypic traits included VB_A, PZ_VB_A, PZ_T, PZ_A, and MOA, to build the prediction models from the micro‐phenotypes to the bending moment. Based on the selected traits, five classic machine learning‐based regression models, including both linear (Ridge regression) and nonlinear approaches (Random Forest, AdaBoost, SVR, and Neural Networks), were trained and evaluated for the prediction performance for the BMMax and BM10 mm. fivefold cross‐validation (the stem data were randomly divided into five groups, where four groups were for training and the remaining group was for testing) was applied for all constructed models. The variable selection, correlation analysis, and regression models were conducted using the Scikit‐learn library (Pedregosa et al., 2012). The root mean square error (RMSE) and the adjusted *R* ^*2*^ score (*R* ^*2*^ adjusted) were used to evaluate the model performance.

### Phenotypic data analysis

The best linear unbiased estimator (BLUP) for each trait in the two environments was estimated using the R language package lme4. The resulting BLUP values were used for subsequent phenotypic analysis. The BLUP calculation model is as follows:

(5) Yikm=μ+gi+τk+gτik+δ(k)m+εikm

where *μ* is the population mean, gi is the genotype effect, *τ*k is the environmental effect, g*τ*ik is the interaction effect of genotype and environment, *δ(k)m* is the effect of the *m*‐th replicate within the *k*‐th environment, and *ε*ikm is the random error effect. Genotype effects were fixed effects, and other factors were random effects.

Microsoft Excel 2019 and GraphPad Prism 8 were used to sort out and analyze the experimental data, and calculate the maximum, minimum, average, standard deviation, etc. Statistical analysis, including variance analysis, correlation analysis, and cluster analysis, along with corresponding visual representations, were performed using Python 3.13, SigmaPlot 14.0, OriginPro 2024, and R 4.3.0.

### Genome‐wide association study

GWAS was conducted on 383 accessions using genotype data available on the MaizeGO website (http://www.maizego.org/Resources.html). PLINK was used to calculate the minor allele frequency (MAF) of each SNP. SNPs with MAF < 0.05 and missingness > 0.1 were filtered out. The remaining effective SNPs were used for subsequent population structure and kinship calculations. To reduce false positives, the first two principal components and the kinship matrix obtained from principal component analysis were included as covariates in the GWAS model (Yu et al., 2006). Multivariate linear mixed models (LMMs) in GEMMA (Genome‐wide Efficient Mixed Model Association algorithm, version 0.98.1) software were used for GWAS analysis (Sun et al., 2021; Jin et al., 2023). A significance threshold of *P* ≤ 1.5446e‐6 was used to identify significant SNP loci. ANNOVAR was used for gene annotation based on the corresponding assembly version. Functional annotation and analysis were performed using the maize B73 reference genome (B73RefGen_v4) from the MaizeGDB database (https://www.maizegdb.org/) and the NCBI database (https://www.ncbi.nlm.nih.gov/). For Zm00001eb416130 (*ZmLSM2*), we combined the syntenic regions of B73RefGen_v4 and B73RefGen_v5 for annotation and used Iso‐seq for verification. Results confirmed that Zm00001d024591 and Zm00001d024593 in B73RefGen_v4 are actually one gene: Zm00001eb416130 (*ZmLSM2*).

Linkage disequilibrium patterns surrounding the significant sites identified by the GWAS were constructed using “LDBlockShow” v1.40 (Dong et al., 2021). The haplotypes were calculated using the “Haplotype Reconstruction (PHAS)” option in DNASP6 software (Rozas et al., 2017).

### Heritability analysis

Heritability refers to the percentage of genetic variation ($V_{A}$) that accounts for the total variation in the phenotype, generally denoted by $H^{2}$. It can be used to compare the relationships between genetic and environmental factors for a specific phenotypic variation. Heritability ($H^{2}$) was calculated for each trait as follows:

(6) $$H^{2}=\frac{V_{g}}{V_{g}+\frac{V_{\mathrm{GL}}}{L}+\frac{V_{e}}{L*R}}$$

where *L* is the number of locations, *R* is the number of replications, and *V*_*g*_, *V*_GL_, and *Ve* represent, respectively, the genotypic variance, interaction between inbred lines and environment variance, and the error variance, respectively. The above analysis was performed in ASReml‐R v.3.0 using the “asreml” function of *R* package asreml (David, 2009).

### Functional identification of the candidate vascular bundle mechanical traits‐related gene

1. Phenotype validation of the *zmlsm2* EMS mutant. The *zmlsm2* EMS mutant (Mut_Sample EMS5‐0ab9e0), in which the glutamine codon CAG at 74 aa is changed into an early stop codon (TAG), was obtained from the Maize EMS‐induced Mutant Database (http://www.elabcaas.cn/memd/index.php) (Lu et al., 2018). The *zmlsm2* EMS mutant was then sequenced to confirm the mutation. The homozygous *zmlsm2* mutant and WT plants were identified and used for detailed phenotyping and further analysis. From June 12 to August 28, 2024, we planted *zmlsm2* and B73 (wild type) for stem and vascular bundle traits' detection. Each genotype was planted at least 15 plants. At the silking stage, stems of all maize plants were selected for a three‐point bending test and CT imaging. Primers for homozygosity identification of maize mutant (EMS5‐0ab9e0) were as follows:

5′‐ATCCCATCCATTGCAGACCG‐3′

5′‐CAGCGTCCGTTACTCCGTTA‐3′

2. Generation of CRISPR–Cas9‐edited mutants of *zmlsm2*. The knockout mutants of *zmlsm2* were produced from a high‐throughput genome‐editing system (Liu et al., 2020). A double sgRNAs' pool approach was used for vector construction. Two target sites were designed as follows:

Target 1: GCGCGTGGGGAGCCTGGCTA CGG

Target 2: GAACTGCTTCATCCGGGGCT CGG

The vector (pEGOsCas9Pubi‐B‐origin) used in this study came from Aoyu Biotechnology, with modifications. Primers for amplification of the sgRNA expression cassettes were as follows:

Cas‐9‐gRT1: CGCGTGGGGAGCCTGGCTA gttttagagctagaaat

Cas‐9‐OsU6aT1: TAGCCAGGCTCCCCACGCG Cggcagccaagccagca

Cas‐9‐gRT2: AACTGCTTCATCCGGGGCT gttttagagctagaaat

Cas‐9‐OsU6bT2: AGCCCCGGATGAAGCAGTT Caacacaagcggcagc

U‐F: CTCCGTTTTACCTGTGGAATCG

gRNA‐R: CGGAGGAAAATTCCATCCAC

Pps‐R: TTCAGAggtctcT accg ACTAGTATGGAATCGGCAGCAAAGG

Pgs‐L: AGCGTGggtctcG ctcg ACGCGTATCCATCCACTCCAAGCTC

SP‐L: GCGGTGTCATCTATGTTACTAG

SP‐R: TGCAATAACTTCGTATAGGC

The constructed gene‐editing vectors were transformed into maize inbred line B73 using an Agrobacterium‐mediated transformation system.

3. Phenotype validation of *zmlsm2* CRISPR‐Cas9‐edited mutants. Homozygous gene‐edited plant *zmlsm2* and WT plants were identified and used for detailed phenotyping and further analysis. Gene‐edited plants, together with their wild‐type plants, were planted for phenotype validation at the greenhouse of the Beijing Academy of Agriculture and Forestry Sciences from August to December 2024. Maize seeds of uniform size, fullness, and without disease spots were selected and sown in plastic pots (diameter of 30 cm and height of 35 cm) filled with normal soil. Each genotype was planted with at least 15 pots. At the 12‐leaf stage (V12) and the maturity stage (R6), stems of maize plants were selected for a three‐point bending test and CT imaging.

### Library preparation and sequencing

For B73, ZZ01, and CIMBL86, samples were collected at the 7‐leaf stage of maize. Specifically, the third internode tissue from the base of the stalk was excised, spanning from 0.5 cm below the node to 0.5 cm above the next node (Figure S13). Three biological replicates were collected for each sample. The samples were frozen in liquid nitrogen immediately and stored at −80°C until use.

The standard CTAB method was used to prepare DNA for WGBS. Libraries were constructed using 1 μg of genomic DNA. Library quality was checked using Qubit and gel electrophoresis. Sequencing was performed on Nova‐PE150 at Novogene (China). RNA was extracted using the RNeasy Plus Mini Kit (Vazyme Biotech, China). The cDNA libraries were generated using the NEBNext Ultra RNA Library Prep Kit (NEB, USA). The quantified libraries were prepared for sequencing on the Illumina NovaSeq. 6000 (illumina, USA) at Novogene (China). Gene expression levels were calculated with featureCounts (Liao et al., 2014).

### Analysis of WGBS data

For WGBS data analysis, raw reads were aligned to the reference genome using BSMAP v2.90 (Xi and Li, 2009) with the following parameters: –w 1 (to report the best hit) and –n 1 (to align reads to both strands). Methylation levels were quantified using the methratio.py script from the BSMAP package, which extracts the exact number of methylated cytosines at a single‐base‐pair resolution. Low‐quality alignments with a Phred score (Q) < 30 were filtered out using SAMtools v1.12 (Li et al., 2009). PCR duplicates were removed using MarkDuplicates.jar, a Java program from the Picard Toolkit v2.25.6. Broad Institute, GitHub Repository, available at: https://broadinstitute.github.io/picard/). Differentially methylated regions were identified using Metilene v0.2‐8 (Jühling et al., 2016) with the following criteria: Each DMR was required to contain at least eight cytosine sites and have a Q‐value < 0.01 (Bonferroni‐corrected). For CG, CHG, and CHH methylation contexts, differences between two samples were considered statistically significant if they exceeded 0.4, 0.4, and 0.2, respectively. DMR‐associated genes were defined as those containing DMRs within 2 kb.

## CONFLICTS OF INTEREST

The authors declare no conflicts of interest.

## AUTHOR CONTRIBUTIONS

C.Z. and X.G. conceptualized this study and revised the manuscript. Y.Z., Z.W., J.D., J.L., Q.L., and G.H. drafted and revised the manuscript. X.G., Y.Z., J.D., Y.Z., Y.W., Q.M., M.G., M.Z., C.W., and X.L. performed field experiments and obtained experimental data. J.D. developed the image‐processing pipeline. Y.Z., Y.Z., Y.W., and Z.W. carried out the statistical analysis and GWAS work. Y.Z., Y.Z., and Q.M. conducted functional verification of candidate genes. Q.L., Z.W., and J.L. conducted the WGBS work and analyzed WGBS and RNA‐seq data. All authors read and approved the final manuscript.

## Supporting information

Additional Supporting Information may be found online in the supporting information tab for this article: http://onlinelibrary.wiley.com/doi/10.1111/jipb.70140/suppinfo

**Figure S1.** Accuracy evaluation for five quantity‐related and size‐related traits of vascular bundles and stem slice
**Figure S2.** Comparison of different microanatomical traits among three maize phenotypic groups
**Figure S3.** Proportion of each clustering group in different‐region subgroups of maize association mapping panel
**Figure S4**. The plot shows the linear regression value between each of the 32 microanatomical traits with BMMax
**Figure S5.** The plot shows the linear regression value between each of the 32 microanatomical traits with BM10 mm
**Figure S6.** Correlation analysis and recursive feature elimination for microanatomical traits and BM10 mm
**Figure S7.** Correlation analysis and recursive feature elimination for microanatomical traits and BMMax
**Figure S8.** Influence of the *zmlsm2* EMS mutation on maize stem development
**Figure S9.** Influence of the *zmlsm2* EMS mutation on maize stem microanatomical traits and mechanical properties
**Figure S10.** Comparison of stem cross‐section CT images between *zmlsm2* knockout mutants and B73 wild‐type plants
**Figure S11.** Expression level of the *ZmLSM2* gene in the *zmlsm2* knockout mutant and WT
**Figure S12.** The *zmlsm2* knockout mutants caused microanatomical trait changes in stem and vascular bundles and stem mechanical properties in maize in the greenhouse at the R6 stage
**Figure S13.** The third internode tissue of stems from the V7 stage (B73, ZZ01, and CIMBL86) was used for WGBS and RNA sequencing
**Figure S14.** Micro‐CT scanning shows the stem cross‐section structure of the third internodes of B73, ZZ01, and CIMBL86 at the silking stage
**Figure S15.** Comparison of microanatomical traits between B73, ZZ01, and CIMBL86
**Figure S16.** Principal component analysis showing the correlation between biological replicates of whole‐genome bisulfite sequencing samples
**Figure S17.** Visualization of *ZmLSM2* transcriptional patterns and splicing events across different maize inbred lines

**Table S1.** Information of 383 maize accessions in the panel
**Table S2.** Microanatomical traits of stem and vascular bundles' description and abbreviation
**Table S3.** List of the inbred lines corresponding to the CT image number
**Table S4.** Population clustering results of maize association based on stem microanatomical BLUP values
**Table S5.** Phenotypic variations of 32 traits of the third internode of maize stem among the natural population
**Table S6.** Microanatomical traits of stem and vascular bundles showed signiﬁcant differences among the subpopulations of 383 inbred lines (TST, NSS, SS, and mixed)
**Table S7.** Heritability of the investigated 32 phenotypic traits of maize stem and vascular bundles
**Table S8.** The microanatomical traits of stem and vascular bundles showed signiﬁcant differences among different regional subpopulations of 383 inbred lines (China, CIMMTY, and USA)
**Table S9.** Information of maize material used to build mechanical prediction models
**Table S10.** Summary of trait‐associated SNPs and candidate genes
**Table S11.** Predictive accuracy of models for predicting the bending movement of stems
**Table S12.** Comparison of the stem micro‐phenotype detection method based on CT with the existing paraffin section combined with the optical microscopy method

## Data Availability

CT cross‐section images of the third internode from 383 maize inbred lines grown in Beijing and Sanya during two growing seasons can be downloaded via the link: https://pan.baidu.com/s/1CP2kkAmTvy1zi3QJGtKSWQ?pwd=JIPB. Extraction code: JIPB. The whole‐genome methylation data and transcriptome data for three maize cultivars (B73, ZZ01, and CIMBL86) have been deposited in the Sequence Read Archive (SRA) database under the accession number PRJNA1254795. The data can be accessed via the following reviewer link: https://dataview.ncbi.nlm.nih.gov/object/PRJNA1254795?reviewer=j24bqrqs0742uisrogiee74c4g. All other reasonable requests for data and research materials are available by contacting the corresponding authors.

## References

[jipb70140-bib-0001] Cao, S. , Chen, K. , Lu, K. , Chen, S. , Zhang, X. , Shen, C. , Zhu, S. , Niu, Y. , Fan, L. , Chen, Z.J. , et al. (2023). Asymmetric variation in DNA methylation during domestication and de‐domestication of rice. Plant Cell 35: 3429–3443. 37279583 10.1093/plcell/koad160 PMC10473196

[jipb70140-bib-0002] Cook, D.D. , Meehan, K. , Asatiani, L. , and Robertson, D.J. (2020). The effect of probe geometry on rind puncture resistance testing of maize stalks. Plant Methods 16: 65. 32411274 10.1186/s13007-020-00610-8 PMC7206738

[jipb70140-bib-0003] Costa, F.M. , Vidal, R. , de Almeida Silva, N.C. , Veasey, E.A. , de Oliveira Freitas, F. , and Zucchi, M.I. (2024). Archaeological findings show the extent of primitive characteristics of maize in South America. Sci. Adv. 10: eadn1466. 39231236 10.1126/sciadv.adn1466 PMC11373604

[jipb70140-bib-0004] David, B. (2009). Asreml‐Fit the Linear Mixed Model, R package version 3.0. VSN International Ltd, Hemel Hempstead. Hemel Hempstead, UK.

[jipb70140-bib-0005] De Rybel, B. , Mähönen, A.P. , Helariutta, Y. , and Weijers, D. (2016). Plant vascular development: From early specification to differentiation. Nat. Rev. Mol. Cell Biol. 17: 30–40. 26580717 10.1038/nrm.2015.6

[jipb70140-bib-0006] Doebley, J. (2004). The genetics of maize evolution. Annu. Rev. Genet. 38: 37–59. 15568971 10.1146/annurev.genet.38.072902.092425

[jipb70140-bib-0007] Doebley, J. , Stec, A. , and Hubbard, L. (1997). The evolution of apical dominance in maize. Nature 386: 485–488. 9087405 10.1038/386485a0

[jipb70140-bib-0008] Dong, S.S. , He, W.M. , Ji, J.J. , Zhang, C. , Guo, Y. , and Yang, T.L. (2021). LDBlockShow: A fast and convenient tool for visualizing linkage disequilibrium and haplotype blocks based on variant call format files. Brief. Bioinform. 22: bbaa227. 33126247 10.1093/bib/bbaa227

[jipb70140-bib-0009] Du, J. , Zhang, Y. , Lu, X. , Zhang, M. , Wang, J. , Liao, S. , Guo, X. , and Zhao, C. (2022). A deep learning‐integrated phenotyping pipeline for vascular bundle phenotypes and its application in evaluating sap flow in the maize stem. Crop J. 10: 1424–1434.

[jipb70140-bib-0010] FAO, IFAD, UNICEF, WFP, and WHO . (2021). The State of Food Security and Nutrition in the World 2021. Transforming food systems for food security, improved nutrition and affordable healthy diets for all. FAO, Rome, Italy.

[jipb70140-bib-0011] Guo, H. , Cao, P. , Wang, C. , Lai, J. , Deng, Y. , Li, C. , Hao, Y. , Wu, Z. , Chen, R. , Qiang, Q. , et al. (2023). Population analysis reveals the roles of DNA methylation in tomato domestication and metabolic diversity. Sci. China Life Sci. 66: 1888–1902. 36971992 10.1007/s11427-022-2299-5

[jipb70140-bib-0012] Hu, Y. , Zhao, T. , Guo, Y. , Wang, M. , Brachhold, K. , Chu, C. , Hanson, A. , Kumar, S. , Lin, R. , Long, W. , et al. (2023). 100 essential questions for the future of agriculture. Mod. Agric. 1: 4–12.

[jipb70140-bib-0013] Huang, C. , Chen, Q. , Xu, G. , Xu, D. , Tian, J. , and Tian, F. (2016). Identification and fine mapping of quantitative trait loci for the number of vascular bundle in maize stem. J. Integr. Plant Biol. 58: 81–90. 25845500 10.1111/jipb.12358 PMC5034846

[jipb70140-bib-0014] Huang, G. , Li, Y. , Zhang, Y. , Wen, W. , Zhao, C. , and Guo, X. (2025). Overcoming challenges in plant biomechanics: Methodological innovations and technological integration. Advanced Sci. (Weinh) 12: e2415606. 10.1002/advs.202415606 PMC11904986 39887899

[jipb70140-bib-0015] Jin, Y. , Wang, J. , Zhang, Y. , Zhao, Y. , Lu, X. , Wen, W. , Liu, X. , Guo, X. , and Zhao, C. (2023). Differential analysis and genome‐wide association analysis of stomata density of maize inbred lines leaves at ear position. Can. J. Plant Sci. 103: 529–540.

[jipb70140-bib-0016] Jühling, F. , Kretzmer, H. , Bernhart, S.H. , Otto, C. , Stadler, P.F. , and Hoffmann, S. (2016). metilene: Fast and sensitive calling of differentially methylated regions from bisulfite sequencing data. Genome Res. 26: 256–262. 26631489 10.1101/gr.196394.115 PMC4728377

[jipb70140-bib-0017] Kawakatsu, T. , Huang, S.C. , Jupe, F. , Sasaki, E. , Schmitz, R.J. , Urich, M.A. , Castanon, R. , Nery, J.R. , Barragan, C. , He, Y. , et al. (2016). Epigenomic diversity in a global collection of *Arabidopsis thaliana* accessions. Cell 166: 492–505. 27419873 10.1016/j.cell.2016.06.044 PMC5172462

[jipb70140-bib-0018] Lev Maor, G. , Yearim, A. , and Ast, G. (2015). The alternative role of DNA methylation in splicing regulation. Trends Genet. 31: 274–280. 25837375 10.1016/j.tig.2015.03.002

[jipb70140-bib-0019] Li, H. , Handsaker, B. , Wysoker, A. , Fennell, T. , Ruan, J. , Homer, N. , Marth, G. , Abecasis, G. , and Durbin, R. (2009). The sequence alignment/map format and SAMtools. Bioinformatics 25: 2078–2079. 19505943 10.1093/bioinformatics/btp352 PMC2723002

[jipb70140-bib-0020] Li, H. , Peng, Z. , Yang, X. , Wang, W. , Fu, J. , Wang, J. , Han, Y. , Chai, Y. , Guo, T. , Yang, N. , et al. (2013). Genome‐wide association study dissects the genetic architecture of oil biosynthesis in maize kernels. Nat. Genet. 45: 43–50. 23242369 10.1038/ng.2484

[jipb70140-bib-0021] Li, J. , Yang, M. , He, D. , Luo, Z. , Li, B. , Huang, X. , Wu, F. , Xie, G. , Fan, C. , Sun, W. , et al. (2024a). Genome‐wide association study of stem structural characteristics that extracted by a high‐throughput phenotypic analysis “LabelmeP rice” in rice. Plant J. 119: 2080–2095. 38860937 10.1111/tpj.16872

[jipb70140-bib-0022] Li, Y.L. , Leu, H.B. , Ting, C.H. , Lim, S.S. , Tsai, T.Y. , Wu, C.H. , Chung, I.F. , and Liang, K.H. (2024b). Predicting long‐term time to cardiovascular incidents using myocardial perfusion imaging and deep convolutional neural networks. Sci. Rep. 14: 3802. 38360974 10.1038/s41598-024-54139-0 PMC10869727

[jipb70140-bib-0023] Liao, Y. , Smyth, G.K. , and Shi, W. (2014). featureCounts: An efficient general purpose program for assigning sequence reads to genomic features. Bioinformatics 30: 923–930. 24227677 10.1093/bioinformatics/btt656

[jipb70140-bib-0024] Liu, H.J. , Jian, L. , Xu, J. , Zhang, Q. , Zhang, M. , Jin, M. , Peng, Y. , Yan, J. , Han, B. , Liu, J. , et al. (2020). High‐throughput CRISPR/Cas9 mutagenesis streamlines trait gene identification in maize. Plant Cell 32: 1397–1413. 32102844 10.1105/tpc.19.00934 PMC7203946

[jipb70140-bib-0025] Lu, X. , Liu, J. , Ren, W. , Yang, Q. , Chai, Z. , Chen, R. , Wang, L. , Zhao, J. , Lang, Z. , Wang, H. , et al. (2018). Gene‐indexed mutations in maize. Mol. Plant 11: 496–504. 29223623 10.1016/j.molp.2017.11.013

[jipb70140-bib-0026] Lucas, W.J. , Groover, A. , Lichtenberger, R. , Furuta, K. , Yadav, S.R. , Helariutta, Y. , He, X.Q. , Fukuda, H. , Kang, J. , Brady, S.M. , et al. (2013). The plant vascular system: Evolution, development and functions. J. Integr. Plant Biol. 55: 294–388. 23462277 10.1111/jipb.12041

[jipb70140-bib-0027] Luo, L. , Qu, Q. , Cao, M. , Zhang, Y. , Sun, Y. , Mao, F. , Chen, J. , Zhu, Y. , Yang, Y. , Liu, H. , et al. (2025). Epigenetic maps of pearl millet reveal a prominent role for CHH methylation in regulating tissue‐specific gene expression. aBIOTECH 6: 394–410. 40994442 10.1007/s42994-025-00243-2 PMC12454808

[jipb70140-bib-0028] Manga‐Robles, A. , Santiago, R. , Malvar, R.A. , Moreno‐González, V. , Fornalé, S. , López, I. , Centeno, M.L. , Acebes, J.L. , Álvarez, J.M. , Caparros‐Ruiz, D. , et al. (2021). Elucidating compositional factors of maize cell walls contributing to stalk strength and lodging resistance. Plant Sci. 307: 110882. 33902850 10.1016/j.plantsci.2021.110882

[jipb70140-bib-0029] Murdy, W.H. (1960). The strengthening system in the stem of maize. Ann. Missouri Bot. Gard. 47: 205–226.

[jipb70140-bib-0030] Muszynska, A. , Guendel, A. , Melzer, M. , Tandron Moya, Y.A. , Röder, M.S. , Rolletschek, H. , Rutten, T. , Munz, E. , Melz, G. , Ortleb, S. , et al. (2021). A mechanistic view on lodging resistance in rye and wheat: A multiscale comparative study. Plant Biotechnol. J. 19: 2646–2661. 34449959 10.1111/pbi.13689 PMC8633492

[jipb70140-bib-0031] Muyle, A.M. , Seymour, D.K. , Lv, Y. , Huettel, B. , and Gaut, B.S. (2022). Gene body methylation in plants: Mechanisms, functions, and important implications for understanding evolutionary processes. Genome Biol. Evol. 14: evac038. 35298639 10.1093/gbe/evac038 PMC8995044

[jipb70140-bib-0032] Niklas, K.J. (1998). A statistical approach to biological factors of safety: Bending and shearing in Psilotum axes. Ann. Bot. 82: 177–187.

[jipb70140-bib-0033] Ou, S. , Su, W. , Liao, Y. , Chougule, K. , Agda, J.R.A. , Hellinga, A.J. , Lugo, C.S.B. , Elliott, T.A. , Ware, D. , Peterson, T. , et al. (2019). Benchmarking transposable element annotation methods for creation of a streamlined, comprehensive pipeline. Genome Biol. 20: 275. 31843001 10.1186/s13059-019-1905-y PMC6913007

[jipb70140-bib-0034] Pedregosa, F. , Varoquaux, G. , Gramfort, A. , Michel, V. , Thirion, B. , Grisel, O. , Blondel, M. , Müller, A. , Nothman, J. , Louppe, G. , et al. (2012). Scikit‐learn: Machine learning in Python. J. Mach. Learn. Res. 12: 2825–2830.

[jipb70140-bib-0035] Perea‐Resa, C. , Hernández‐Verdeja, T. , López‐Cobollo, R. , del Mar Castellano, M. , and Salinas, J. (2012). LSM proteins provide accurate splicing and decay of selected transcripts to ensure normal Arabidopsis development. Plant Cell 24: 4930–4947. 23221597 10.1105/tpc.112.103697 PMC3556967

[jipb70140-bib-0036] Péret, B. , De Rybel, B. , Casimiro, I. , Benková, E. , Swarup, R. , Laplaze, L. , Beeckman, T. , and Bennett, M.J. (2009). Arabidopsis lateral root development: An emerging story. Trends Plant Sci. 14: 399–408. 19559642 10.1016/j.tplants.2009.05.002

[jipb70140-bib-0037] Piovesan, A. , Vancauwenberghe, V. , Van De Looverbosch, T. , Verboven, P. , and Nicolaï, B. (2021). X‐ray computed tomography for 3D plant imaging. Trends Plant Sci. 26: 1171–1185. 34404587 10.1016/j.tplants.2021.07.010

[jipb70140-bib-0038] Rozas, J. , Ferrer‐Mata, A. , Sánchez‐DelBarrio, J.C. , Guirao‐Rico, S. , Librado, P. , Ramos‐Onsins, S.E. , and Sánchez‐Gracia, A. (2017). DnaSP 6: DNA sequence polymorphism analysis of large data sets. Mol. Biol. Evol. 34: 3299–3302. 29029172 10.1093/molbev/msx248

[jipb70140-bib-0039] Shah, D.U. , Reynolds, T.P.S. , and Ramage, M.H. (2017). The strength of plants: Theory and experimental methods to measure the mechanical properties of stems. J. Exp. Bot. 68: 4497–4516. 28981787 10.1093/jxb/erx245

[jipb70140-bib-0040] Shiferaw, B. , Prasanna, B.M. , Hellin, J. , and Bänziger, M. (2011). Crops that feed the world 6. Past successes and future challenges to the role played by maize in global food security. Food Secur. 3: 307–327.

[jipb70140-bib-0041] Stokstad, E. (2023). High hopes for short corn. Science 382: 364–367. 37883569 10.1126/science.adl5302

[jipb70140-bib-0042] Stubbs, C.J. , Larson, R. , and Cook, D.D. (2022). Maize stalk stiffness and strength are primarily determined by morphological factors. Sci. Rep. 12: 720. 35031627 10.1038/s41598-021-04114-w PMC8760316

[jipb70140-bib-0043] Sun, X. , Ren, W. , Wang, P. , Chen, F. , Yuan, L. , Pan, Q. , and Mi, G. (2021). Evaluation of maize root growth and genome‐wide association studies of root traits in response to low nitrogen supply at seedling emergence. Crop J. 9: 794–804.

[jipb70140-bib-0044] Swaminathan, K. , Peterson, K. , and Jack, T. (2008). The plant B3 superfamily. Trends Plant Sci. 13: 647–655. 18986826 10.1016/j.tplants.2008.09.006

[jipb70140-bib-0045] Wang, X. , Shi, Z. , Zhang, R. , Sun, X. , Wang, J. , Wang, S. , Zhang, Y. , Zhao, Y. , Su, A. , Li, C. , et al. (2020). Stalk architecture, cell wall composition, and QTL underlying high stalk flexibility for improved lodging resistance in maize. BMC Plant Biol. 20: 515. 33176702 10.1186/s12870-020-02728-2 PMC7659129

[jipb70140-bib-0046] Wright, S.I. , Bi, I.V. , Schroeder, S.G. , Yamasaki, M. , Doebley, J.F. , McMullen, M.D. , and Gaut, B.S. (2005). The effects of artificial selection on the maize genome. Science 308: 1310–1314. 15919994 10.1126/science.1107891

[jipb70140-bib-0047] Wu, D. , Wu, D. , Feng, H. , Duan, L. , Dai, G. , Liu, X. , Wang, K. , Yang, P. , Chen, G. , Gay, A.P. , et al. (2021). A deep learning‐integrated micro‐CT image analysis pipeline for quantifying rice lodging resistance‐related traits. Plant Commun. 2: 100165. 33898978 10.1016/j.xplc.2021.100165 PMC8060729

[jipb70140-bib-0048] Xi, Y. , and Li, W. (2009). BSMAP: Whole genome bisulfite sequence MAPping program. BMC Bioinformatics 10: 232. 19635165 10.1186/1471-2105-10-232 PMC2724425

[jipb70140-bib-0049] Xu, J. , Chen, G. , Hermanson, P.J. , Xu, Q. , Sun, C. , Chen, W. , Kan, Q. , Li, M. , Crisp, P.A. , Yan, J. , et al. (2019). Population‐level analysis reveals the widespread occurrence and phenotypic consequence of DNA methylation variation not tagged by genetic variation in maize. Genome Biol. 20: 243. 31744513 10.1186/s13059-019-1859-0 PMC6862797

[jipb70140-bib-0050] Xue, Y. , Cao, X. , Chen, X. , Deng, X. , Deng, X.W. , Ding, Y. , Dong, A. , Duan, C.G. , Fang, X. , Gong, L. , et al. (2025). Epigenetics in the modern era of crop improvements. Sci. China Life Sci. 68: 1570–1609. 39808224 10.1007/s11427-024-2784-3

[jipb70140-bib-0051] Yang, X. , Gao, S. , Xu, S. , Zhang, Z. , Prasanna, B.M. , Li, L. , Li, J. , and Yan, J. (2010). Characterization of a global germplasm collection and its potential utilization for analysis of complex quantitative traits in maize. Mol. Breed. 28: 511–526.

[jipb70140-bib-0052] Yu, J. , Pressoir, G. , Briggs, W.H. , Vroh Bi, I. , Yamasaki, M. , Doebley, J.F. , McMullen, M.D. , Gaut, B.S. , Nielsen, D.M. , Holland, J.B. , et al. (2006). A unified mixed‐model method for association mapping that accounts for multiple levels of relatedness. Nat. Genet. 38: 203–208. 16380716 10.1038/ng1702

[jipb70140-bib-0053] Zhang, H. , Lang, Z. , and Zhu, J.K. (2018). Dynamics and function of DNA methylation in plants. Nat. Rev. Mol. Cell Biol. 19: 489–506. 29784956 10.1038/s41580-018-0016-z

[jipb70140-bib-0054] Zhang, P. , He, Y. , and Huang, S. (2024a). Unlocking epigenetic breeding potential in tomato and potato. aBIOTECH 5: 507–518. 39650134 10.1007/s42994-024-00184-2 PMC11624185

[jipb70140-bib-0055] Zhang, Y. , Gu, S. , Du, J. , Huang, G. , Shi, J. , Lu, X. , Wang, J. , Yang, W. , Guo, X. , and Zhao, C. (2024b). Plant microphenotype: From innovative imaging to computational analysis. Plant Biotechnol. J. 22: 802–818. 38217351 10.1111/pbi.14244 PMC10955502

[jipb70140-bib-0056] Zhang, Y. , Wang, J. , Du, J. , Zhao, Y. , Lu, X. , Wen, W. , Gu, S. , Fan, J. , Wang, C. , Wu, S. , et al. (2021). Dissecting the phenotypic components and genetic architecture of maize stem vascular bundles using high‐throughput phenotypic analysis. Plant Biotechnol. J. 19: 35–50. 32569428 10.1111/pbi.13437 PMC7769239

[jipb70140-bib-0057] Zhang, Z. , Zhang, X. , Lin, Z. , Wang, J. , Liu, H. , Zhou, L. , Zhong, S. , Li, Y. , Zhu, C. , Lai, J. , et al. (2020). A large transposon insertion in the stiff1 promoter increases stalk strength in maize. Plant Cell 32: 152–165. 31690654 10.1105/tpc.19.00486 PMC6961635

[jipb70140-bib-0058] Zhao, X. , and Zhou, S. (2021). Research progress on traits and assessment methods of stalk lodging resistance in maize. Acta Agron. Sin. 48: 15–26.

[jipb70140-bib-0059] Zhao, B. , Li, K. , Wang, M. , Liu, Z. , Yin, P. , Wang, W. , Li, Z. , Li, X. , Zhang, L. , Han, Y. , et al. (2024). Genetic basis of maize stalk strength decoded via linkage and association mapping. Plant J. 117: 1558–1573. 38113320 10.1111/tpj.16583

[jipb70140-bib-0060] Zheng, Z. , Wang, B. , Zhuo, C. , Xie, Y. , Zhang, X. , Liu, Y. , Zhang, G. , Ding, H. , Zhao, B. , Tian, M. , et al. (2023). Local auxin biosynthesis regulates brace root angle and lodging resistance in maize. New Phytol. 238: 142–154. 36636793 10.1111/nph.18733

