# Supplementary material for: Stem microanatomical phenomic uncovers a potential role for ZmLSM2 in regulating maize stem bending strength
Source: J Integr Plant Biol. 2026 Jan 19;68(5):1271–89. doi: 10.1111/jipb.70140 (PMC13140054; doi:10.1111/jipb.70140)
Supplement: Supplementary file 1 — Figure S1. Accuracy evaluation for five quantity‐related and size‐related traits of vascular bundles and stem slice Figure S2. Comparison of different microanatomical traits among three maize phenotypic groups Figure S3. Proportion of each clustering group in different‐region subgroups of maize association mapping panel Figure S4. The plot shows the linear regression value between each of the 32 microanatomical traits with BMMax Figure S5. The plot shows the linear regression value between each of the 32 microanatomical traits with BM10 mm Figure S6. Correlation analysis and recursive feature elimination for microanatomical traits and BM10 mm Figure S7. Correlation analysis and recursive feature elimination for microanatomical traits and BMMax Figure S8. Influence of the zmlsm2 EMS mutation on maize stem development Figure S9. Influence of the zmlsm2 EMS mutation on maize stem microanatomical traits and mechanical properties Figure S10. Comparison of stem cross‐section CT images between zmlsm2 knockout mutants and B73 wild‐type plants Figure S11. Expression level of the ZmLSM2 gene in the zmlsm2 knockout mutant and WT Figure S12. The zmlsm2 knockout mutants caused microanatomical trait changes in stem and vascular bundles and stem mechanical properties in maize in the greenhouse at the R6 stage Figure S13. The third internode tissue of stems from the V7 stage (B73, ZZ01, and CIMBL86) was used for WGBS and RNA sequencing Figure S14. Micro‐CT scanning shows the stem cross‐section structure of the third internodes of B73, ZZ01, and CIMBL86 at the silking stage Figure S15. Comparison of microanatomical traits between B73, ZZ01, and CIMBL86 Figure S16. Principal component analysis showing the correlation between biological replicates of whole‐genome bisulfite sequencing samples Figure S17. Visualization of ZmLSM2 transcriptional patterns and splicing events across different maize inbred lines [file JIPB-68-1271-s002.docx]

**Extensive stem microanatomical phenomics: uncovers *ZmLSM2* in regulating maize stem bending strength**

Ying Zhang^1,3,4,6,#^, Zejia Wang^2,#^, Jianjun Du^1,3,4,#^, Jiawen Li^2,#^, Guanmin Huang^1,3,4^, Yanxin Zhao^5^, Yanru Wang^1,3,4^, Qingmei Men^1,3,4^, Minkun Guo^1,3,4^, Minggang Zhang^1,3,4^, Xianju Lu^1,3,4^, Chuanyu Wang^1,3,4^, Qikun Liu^2,*^, Xinyu Guo ^1,3,4,*^, Chunjiang Zhao^1,3,4,*^

^*^Correspondence: Qikun Liu (qikunliu@pku.edu.cn), Xinyu Guo (guoxy73@163.com), and Chunjiang Zhao (zhaocj@nercita.org.cn, Prof. Zhao fully responsible for the distribution of all materials associated with this article)


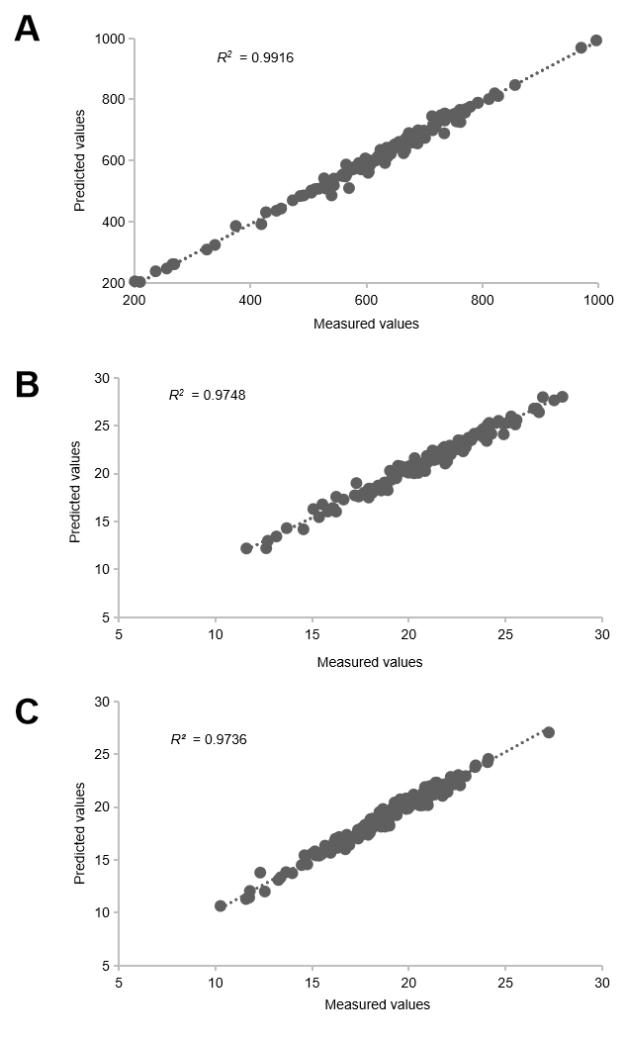


**Figure S1.** **Accuracy evaluation for five quantity-related and size-related traits of vascular bundles and stem slice.**

**(A)** Total number of vascular bundles in stem slice (VB_N). **(B)** Long axis length of the stem slice (SZ_LA). **(C)** Short axis length of the stem slice (SZ_SA) (*n* = 156).


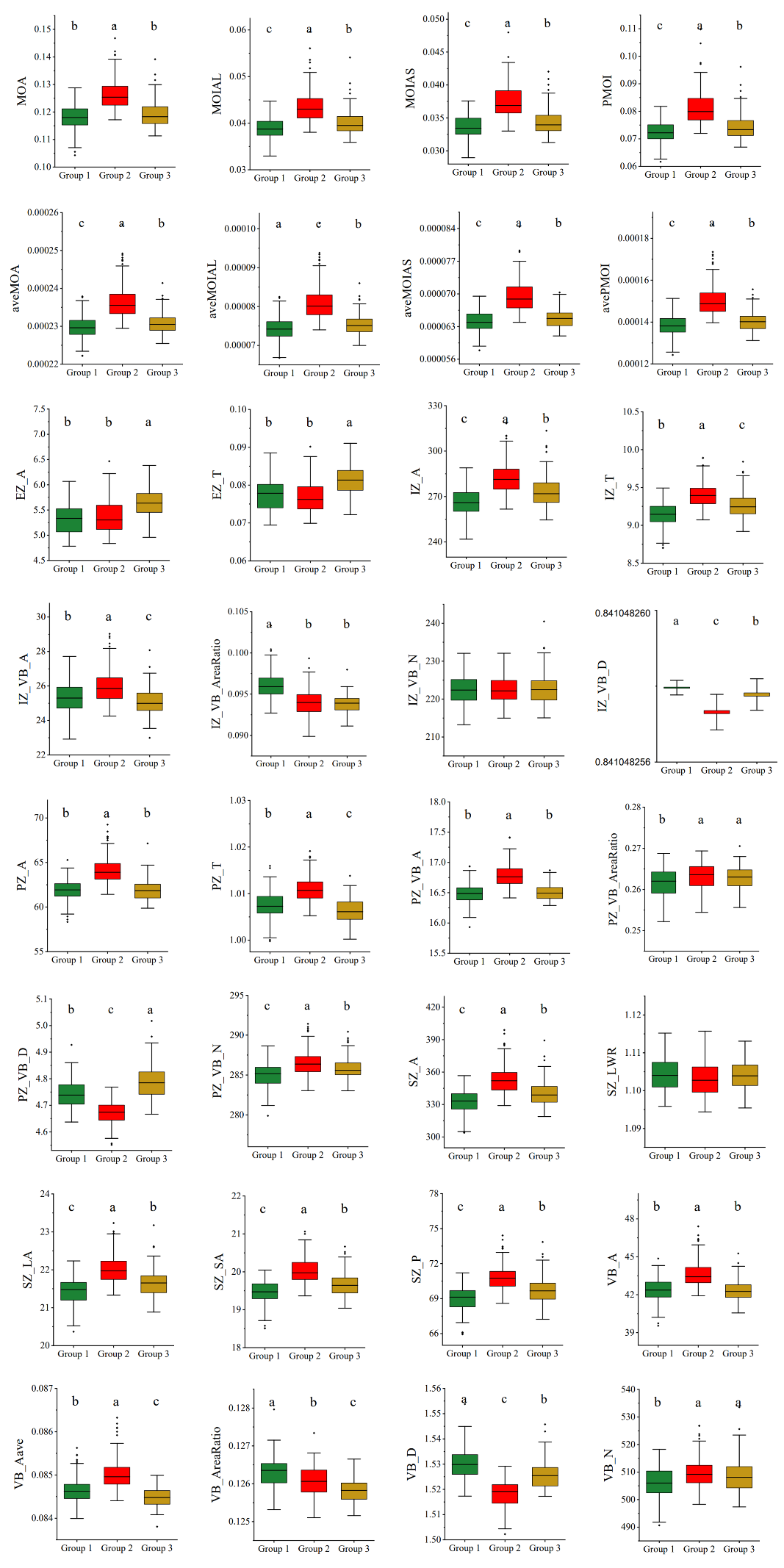


**Figure S2. Comparison of different microanatomical traits among three maize phenotypic groups.**


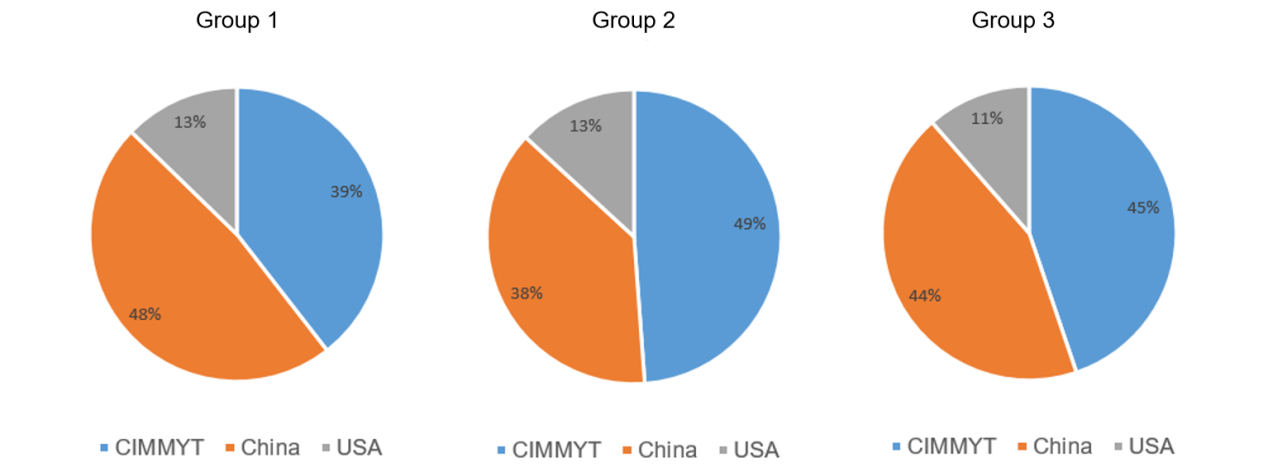


**Figure S3. Proportion of each clustering group in different-region subgroups of maize association mapping panel.**


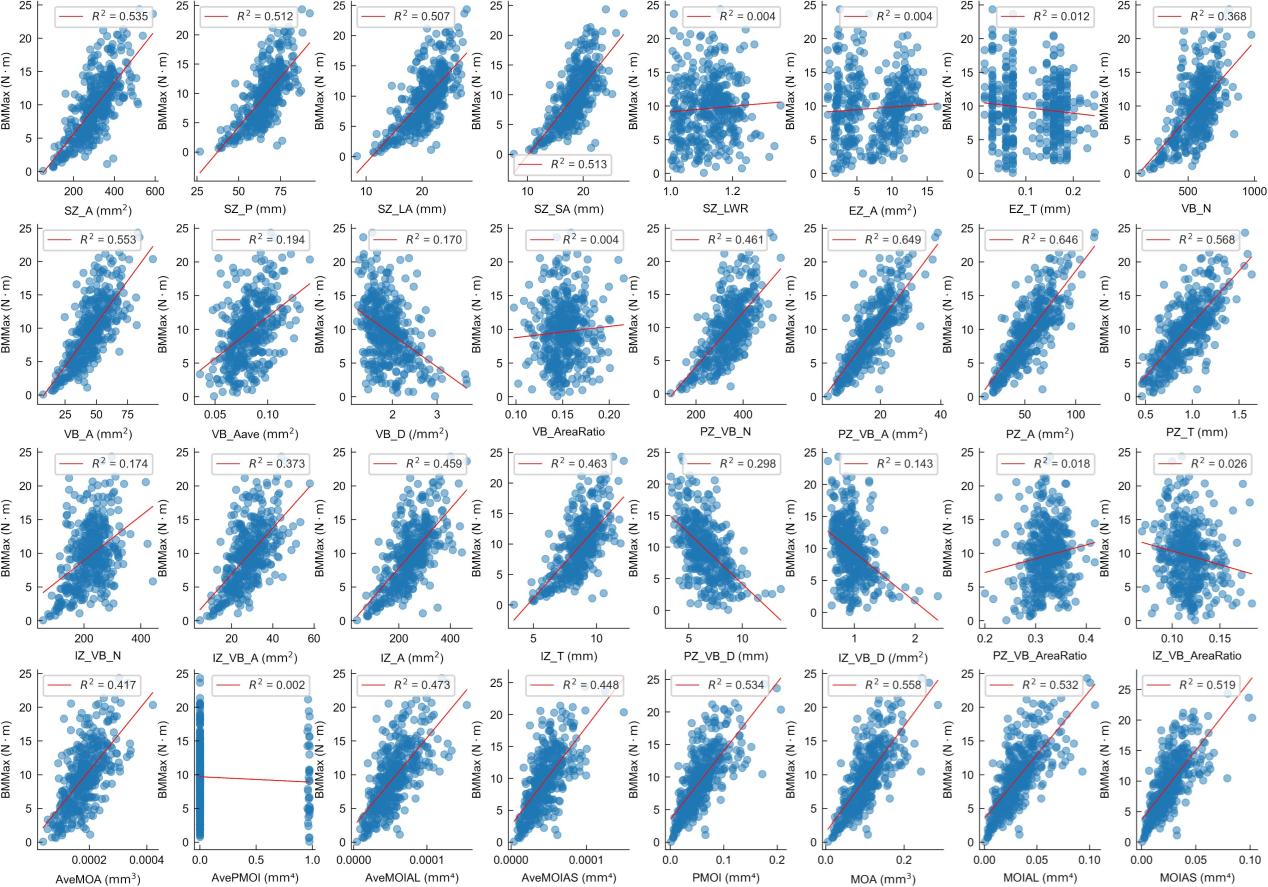


**Figure S4. The plot shows linear regression value between each of the 32 microanatomical traits with BMMax.**

Each dot represents a sample measurement. The red line indicates the linear regression fit.


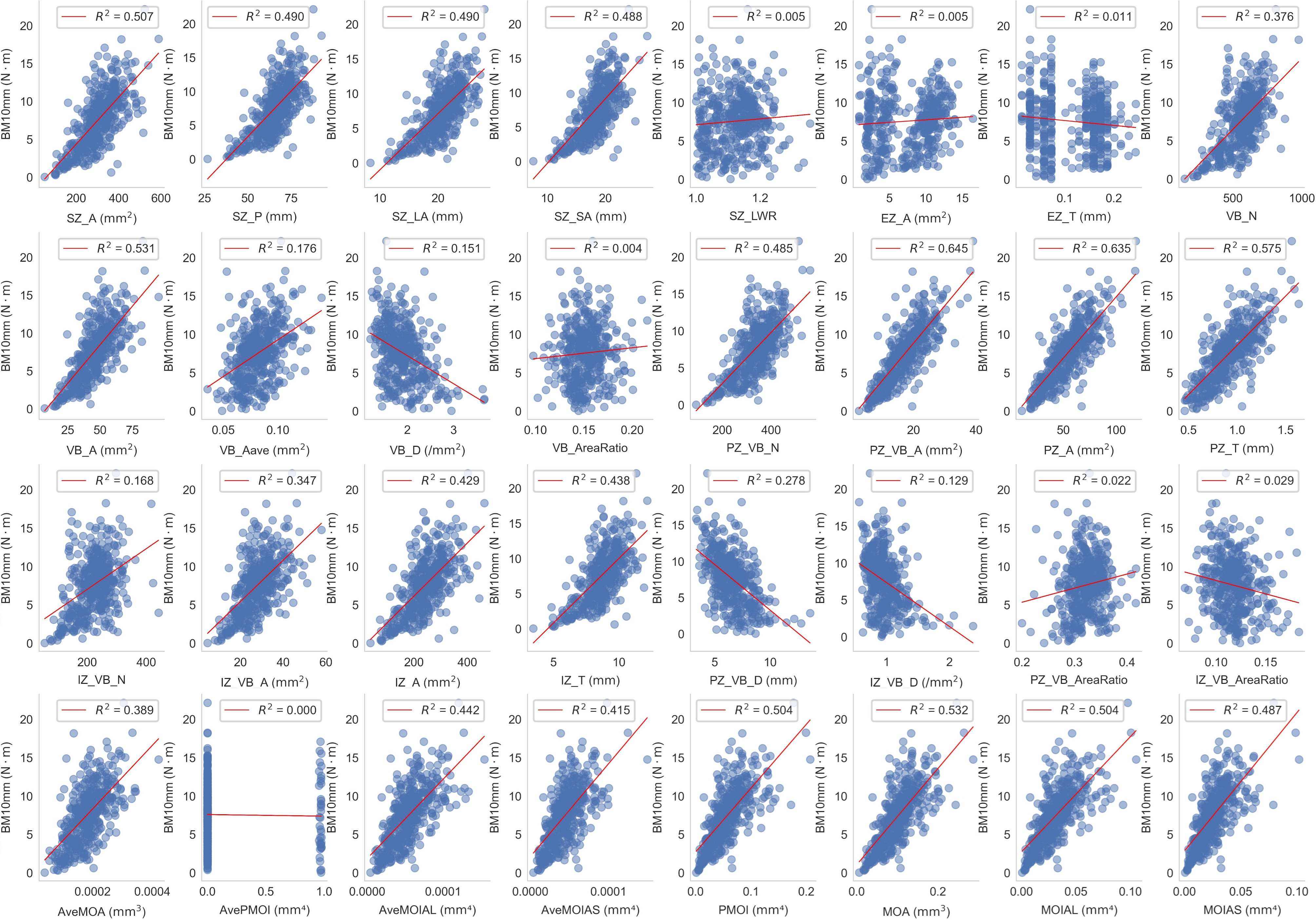


**Figure S5. The plot shows linear regression value between each of the 32 microanatomical traits with BM10 mm.**

Each dot represents a sample measurement. The red line indicates the linear regression fit.


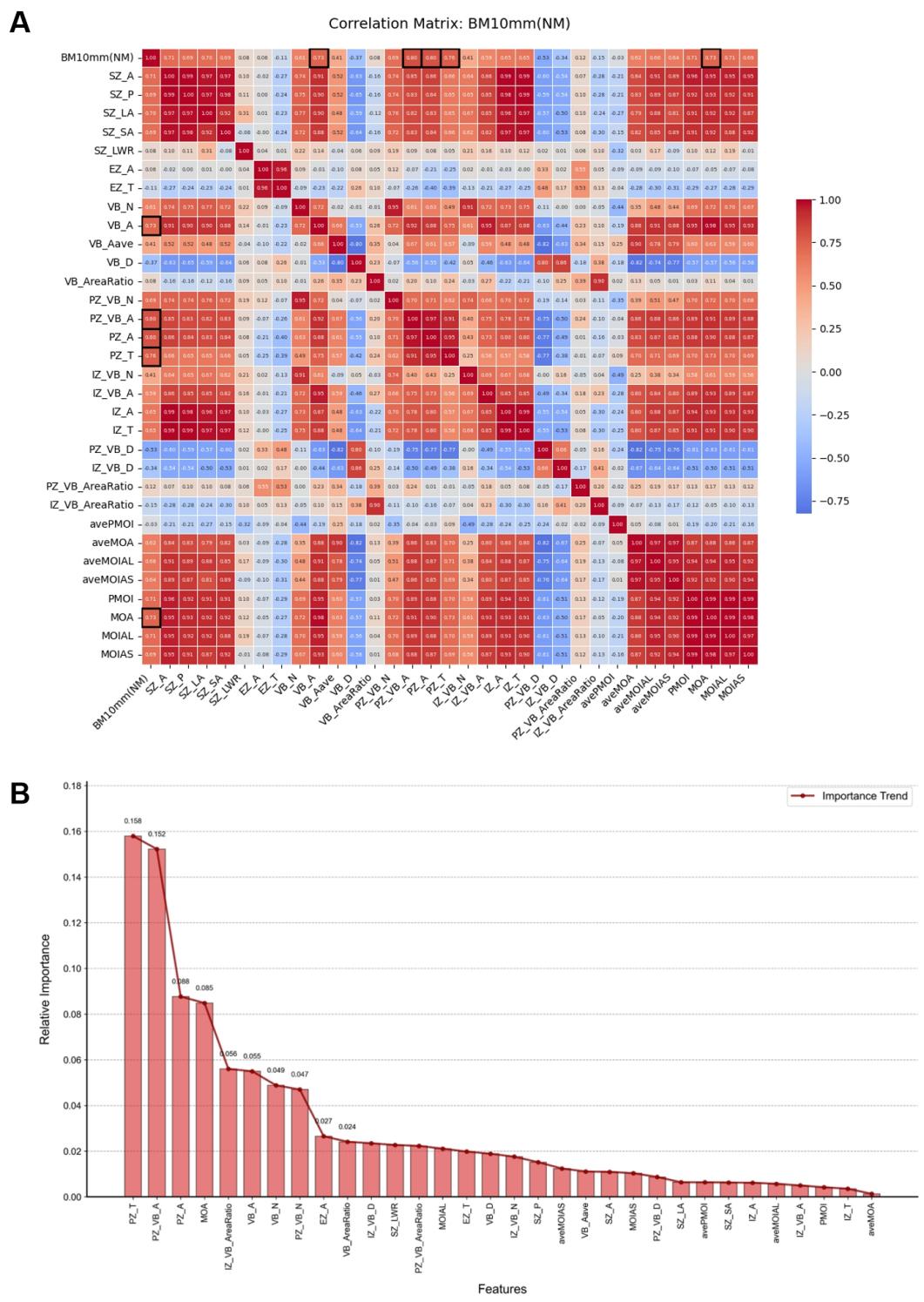


**Figure S6. The correlation analysis and recursive feature elimination for microanatomical traits and BM10 mm.**

**(A)** The correlation analysis for the 32 microanatomical traits and BM10 mm for the third internodes of maize stems. Traits with top-5 feature importance are marked in black box, included VB_A, PZ_VB_A, PZ_T, PZ_A, and MOA. **(B)** Feature importance ranking based on recursive feature elimination (RFE) of the 32 microanatomical traits for the BM10 mm. Top-5 feature were PZ_T, PZ_VB_A, PZ_A, MOA, and IZ_VB_AreaRatio.


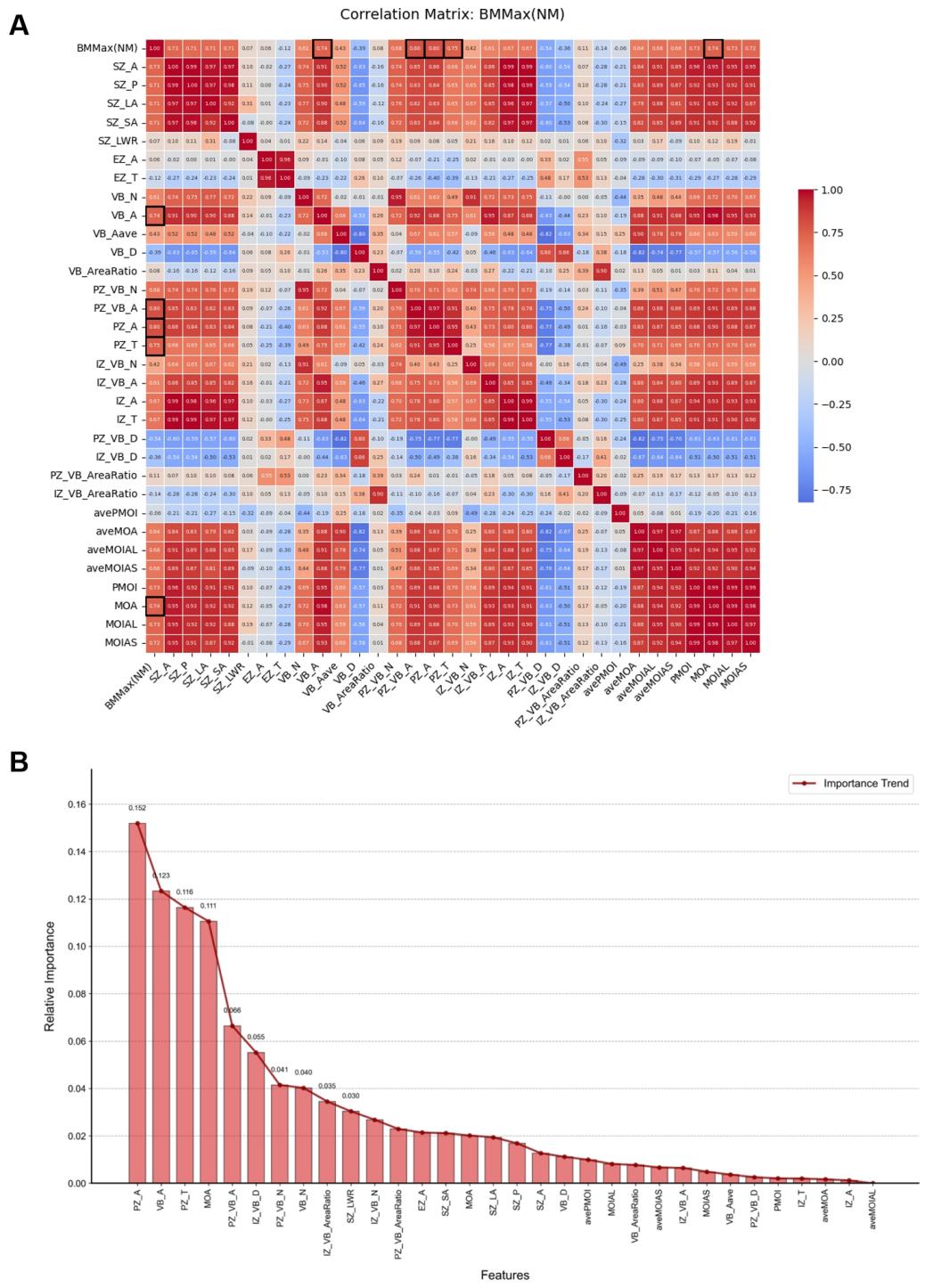


**Figure S7. The correlation analysis and recursive feature elimination for microanatomical traits and BMMax.**

**(A)** The correlation analysis for the 32 microanatomical traits and BMMax for the third internodes of maize stems. Traits with top-5 feature importance are marked in black box, included VB_A, PZ_VB_A, PZ_T, PZ_A, and MOA. **(B)** Feature importance ranking based on recursive feature elimination (RFE) of the 32 microanatomical traits for the BMMax. Top 5 feature were PZ_A, VB_A, PZ_T, MOA, and PZ_VB_A.

**
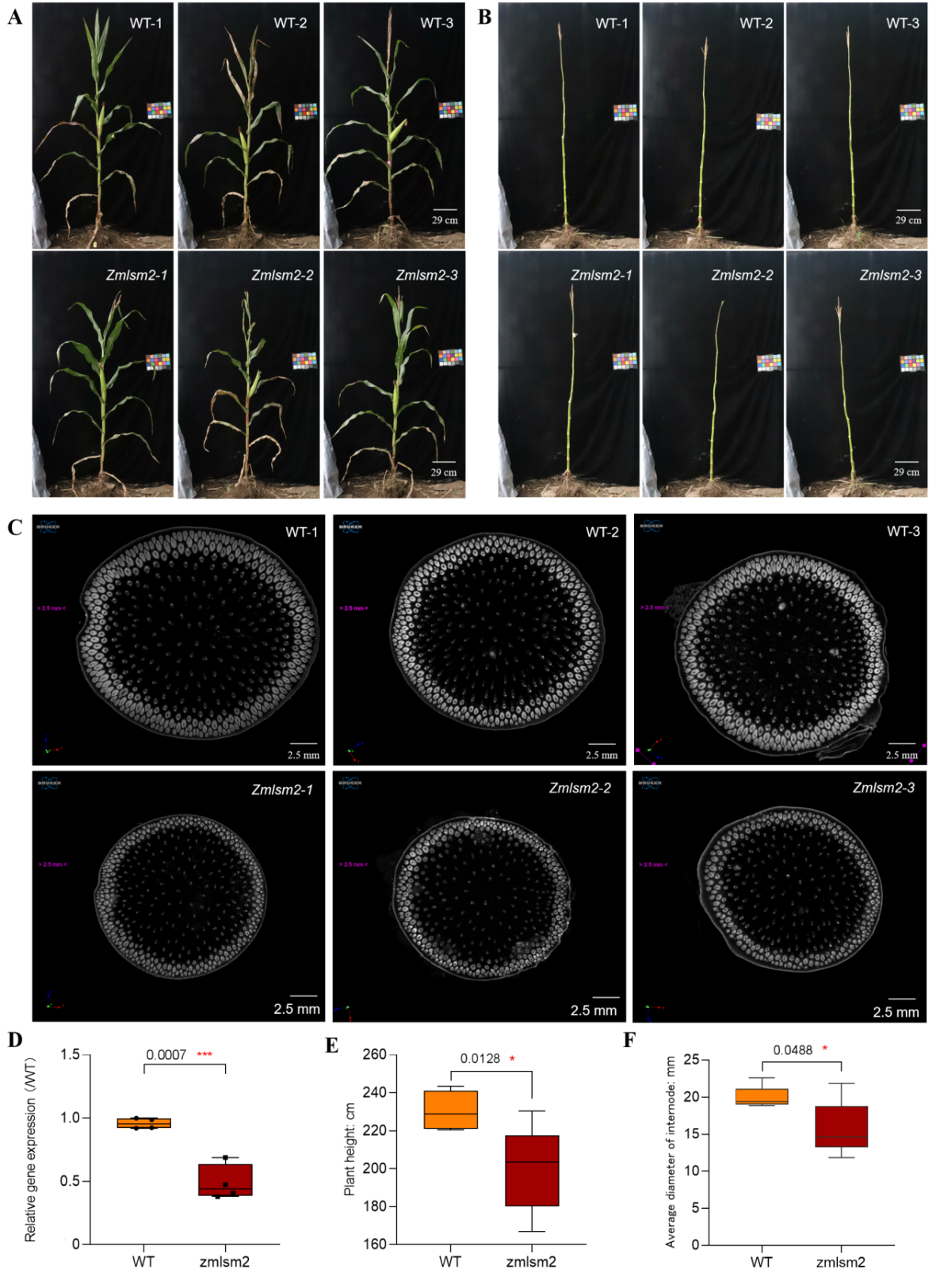
**

**Figure S8. The influence of *zmlsm*2 EMS mutation on maize stem development.**

**(A)** Field-grown WT and *zmlsm2* mutant plants (Scale bar corresponds to 29 cm). **(B)** Stems of field-grown WT and *zmlsm2* mutant plants (Scale bar corresponds to 29 cm). **(C)** The CT scanning images of base internodes from WT and *zmlsm2* mutant plants at the silking stage (Scale bar corresponds to 2.5 mm). **(D)** Expression level of the *ZmLSM2* gene in *zmlsm2* EMS mutant and WT. **(E)** The plant height **(F)** average diameter of internode of the third internodes from *zmlsm2* mutant and WT (B73) maize plants at the silking stage (*n* = 10). Phenotyping of the two groups were compared using Student's *t*-test (* *p* < 0.05, ** *p* < 0.01).


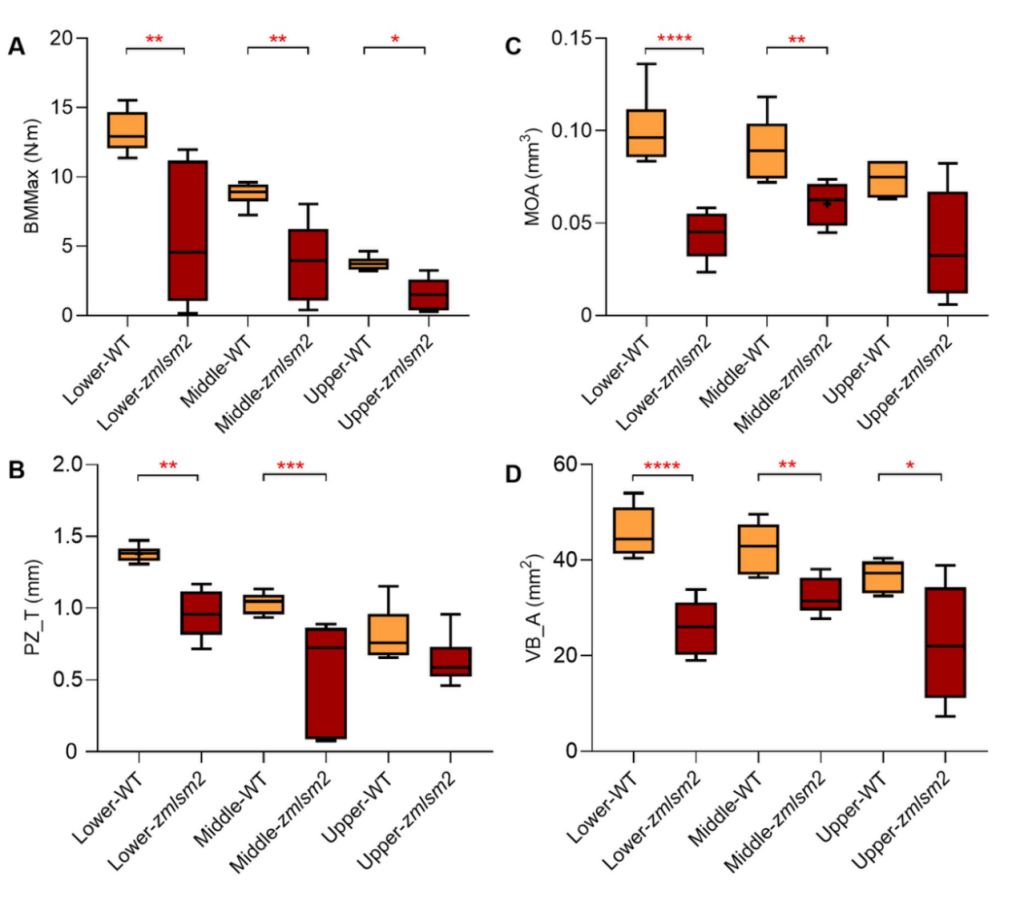


**Figure S9. The influence of *zmlsm2* EMS mutation on maize stem microanatomical traits and mechanical properties.**

**(A)** BMMax, **(B)** PZ_T, **(C)** MOA, and **(D)** VB_A of the lower, middle, and upper internodes from *zmlsm2* mutant and WT maize plants at the silking stage (*n* = 10). Phenotyping of the two groups were compared using Student’s *t*-test (* *p* < 0.05, ** *p* < 0.01, *** 0.001 < *p* < 0.01, **** *p* < 0.001).


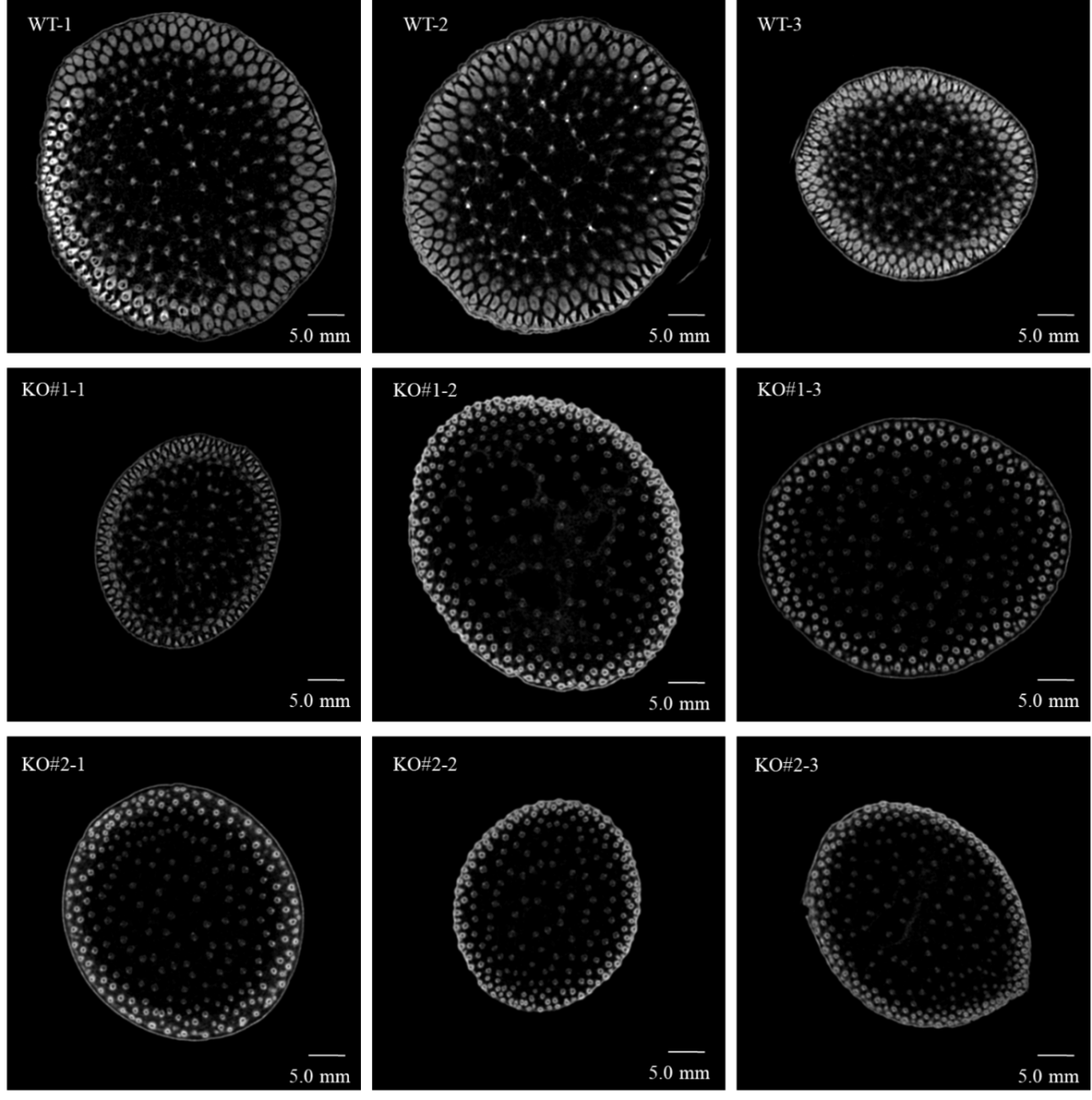


**Figure S10. Comparison of stem cross-section CT images between *zmlsm2* knockout mutants and B73 wild type plants.**

CT scanning images show the base internodes from *zmlsm2* knockout mutants (KO) and B73 wild type (WT) plants grown in the greenhouse at V12 stage (Scale bar corresponds to 5.0 mm).


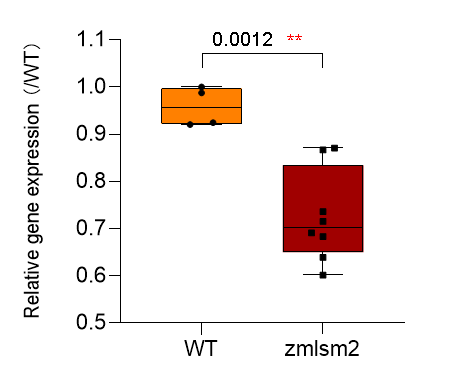


**Figure S11. Expression level of the *ZmLSM2* gene in *zmlsm2* knockout mutant and WT**.

Gene expression of the two groups were compared using Student’s *t*-test (* *p* < 0.05, ** *p* < 0.01, *** *p* < 0.001).


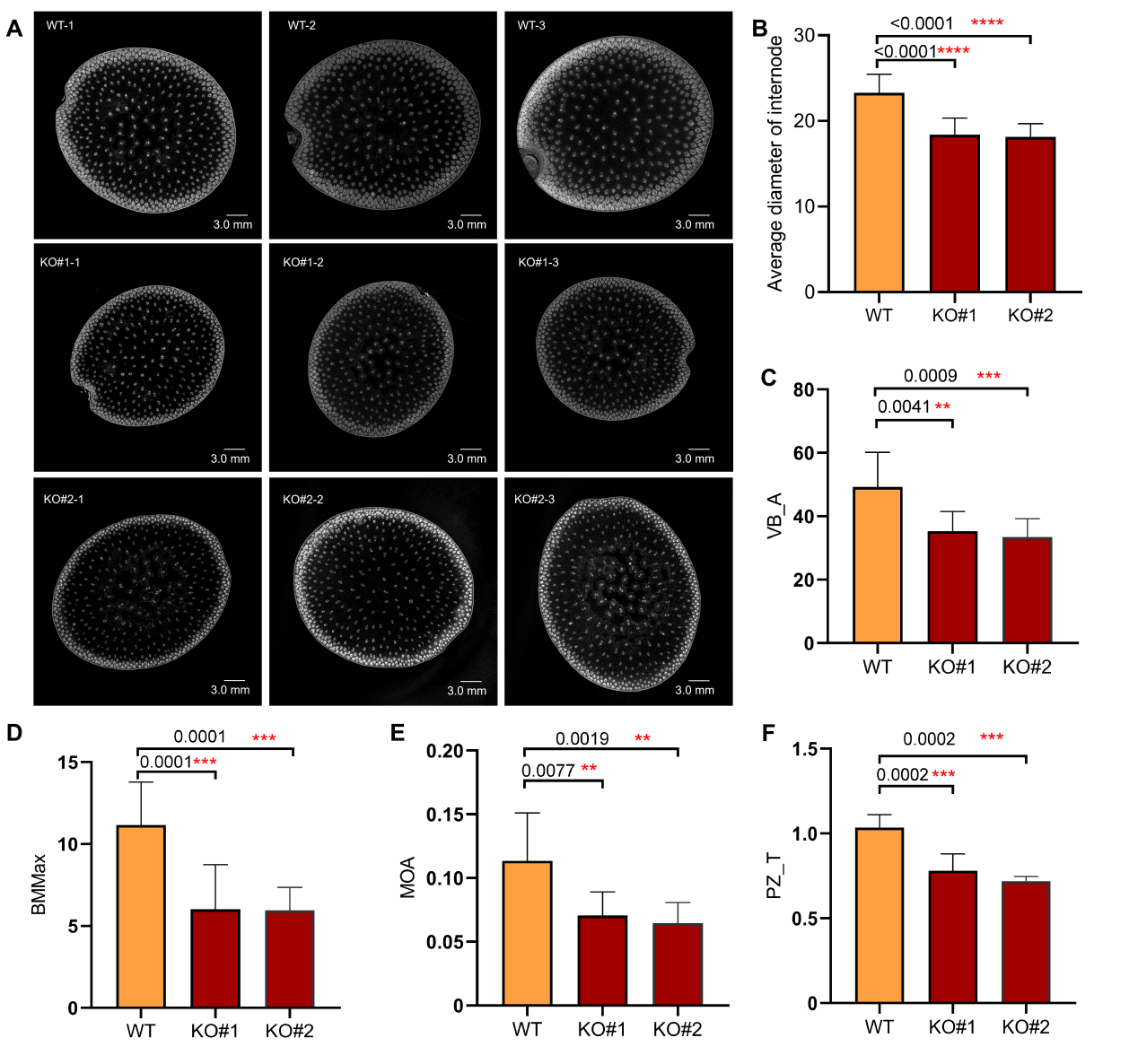


**Figure S12. The *zmlsm2*** **knockout mutants caused the microanatomical trait changes of stem and vascular bundles and stem mechanical properties in maize in the greenhouse** **at R6 stage.**

**(A)** CT scanning images of base internodes from *zmlsm2* knockout mutants (KO) and B73 wild type (WT) plants grown in the greenhouse at R6 stage (Scale bar corresponds to 3.0 mm). **(B)** Average diameter of internode **(C)** VB_A **(D)** BMMax **(E)** MOA and **(F)** PZ_T of the base internodes from *zmlsm2* knockout mutants and WT maize plants at maturity stage (WT, *n* = 10, KO#1, *n* = 8, KO#2, *n* = 8). Phenotyping of the two groups were compared using Student’s *t*-test (* *p* < 0.05, ** *p* < 0.01, *** *p* < 0.001).


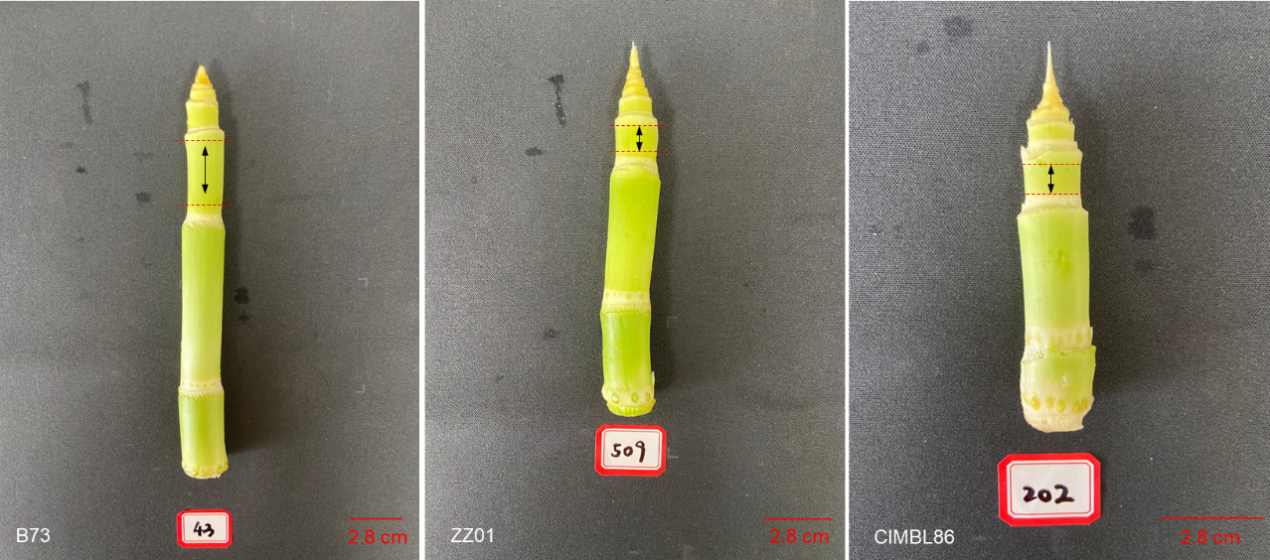


**Figure S13. The third internode tissue of stems from the V7 stage (B73, ZZ01, and CIMBL86) was used for WGBS and RNA sequencing.**

The black arrow indicates the third segment from the base.


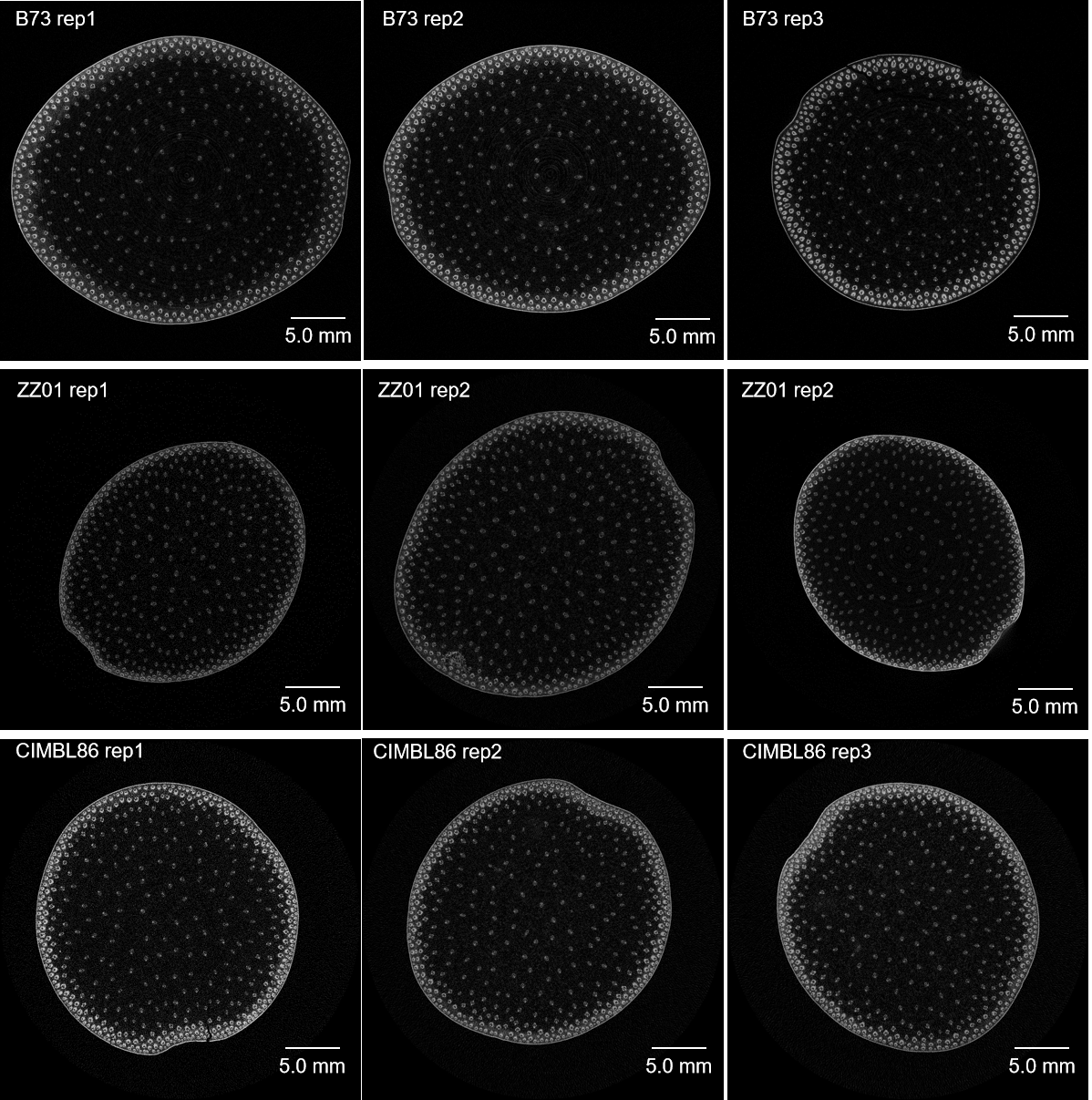


**Figure S14. Micro-CT scanning shows the stem cross-section structure of the third internodes of B73, ZZ01, and CIMBL86 at the silking stage.**

**
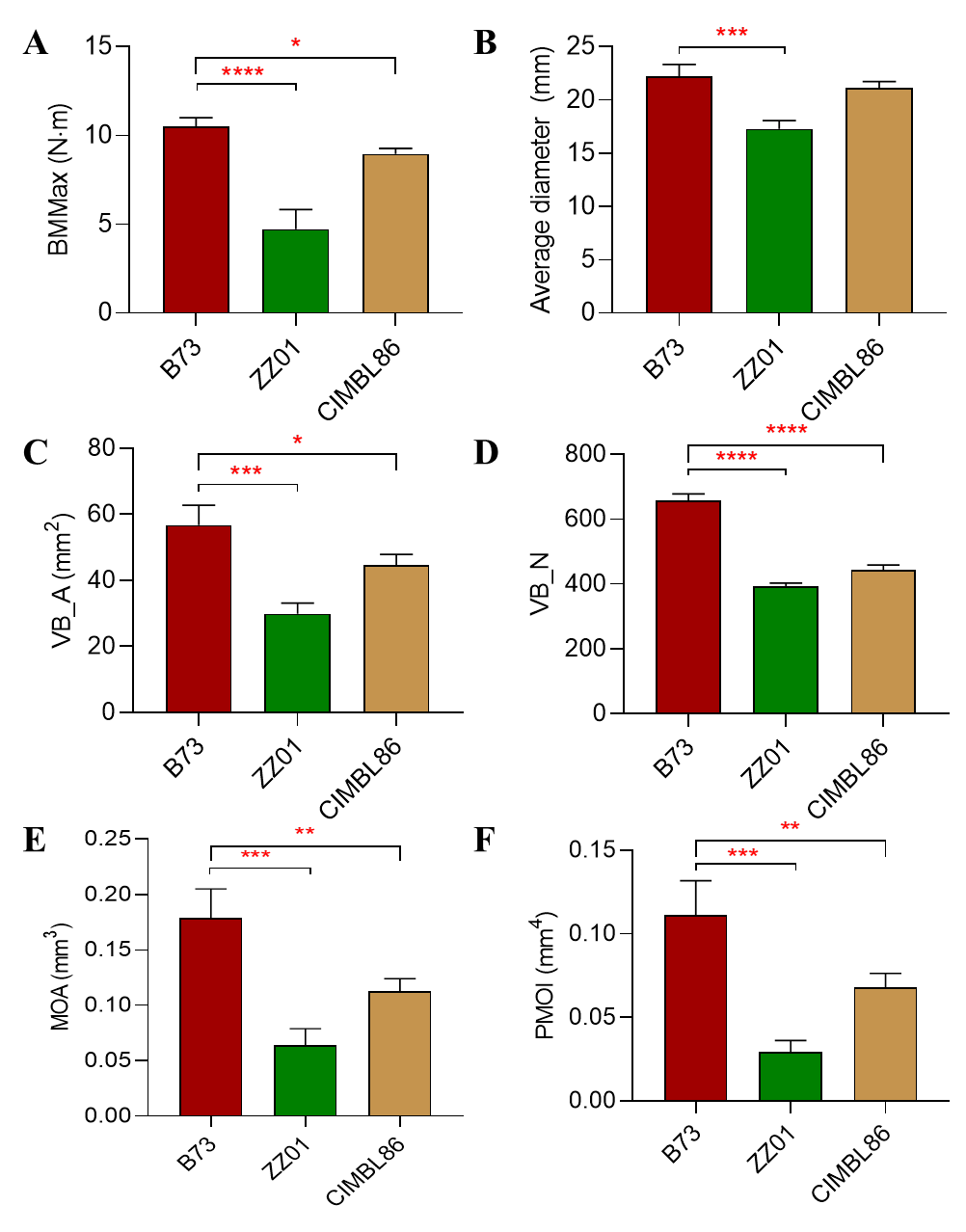
**

**Figure S15. Comparison of microanatomical traits between B73, ZZ01, and CIMBL86.**

Boxplots show **(A)** BMMax, **(B)** Average diameter of internode **(C)** VB_A, **(D)** VB_N, **(E)** MOA and **(F)** PMOI values of B73, ZZ01, and CIMBL86 at the silking stage (*n* = 3). Mann-Whitney-Wilcoxon test, * *p* < 0.05, ** *p* < 0.01, *** *p* < 0.001, **** *p* < 0.0001.

**
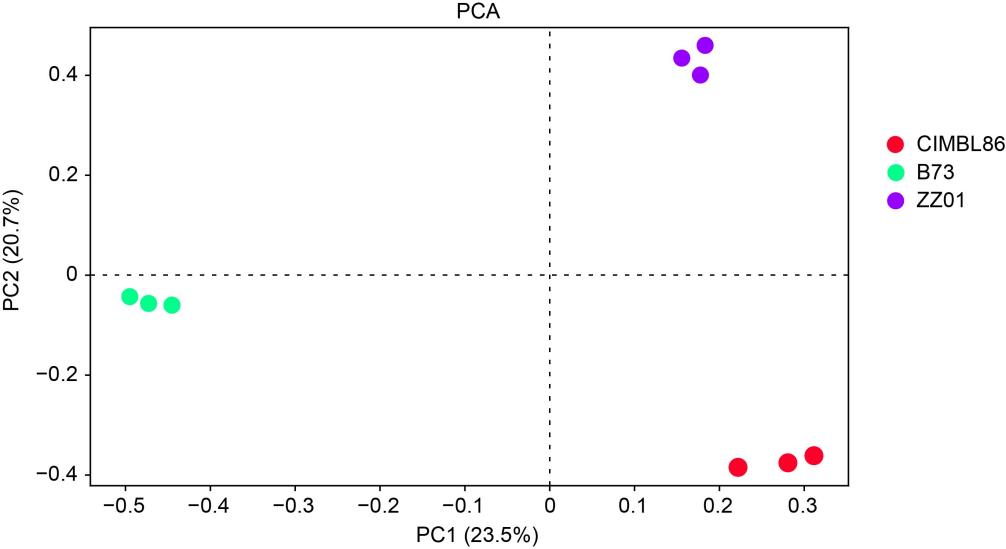
**

**Figure S16. Principal component analysis showing correlation between biological replicates of whole-genome bisulfite sequencing samples.**

**
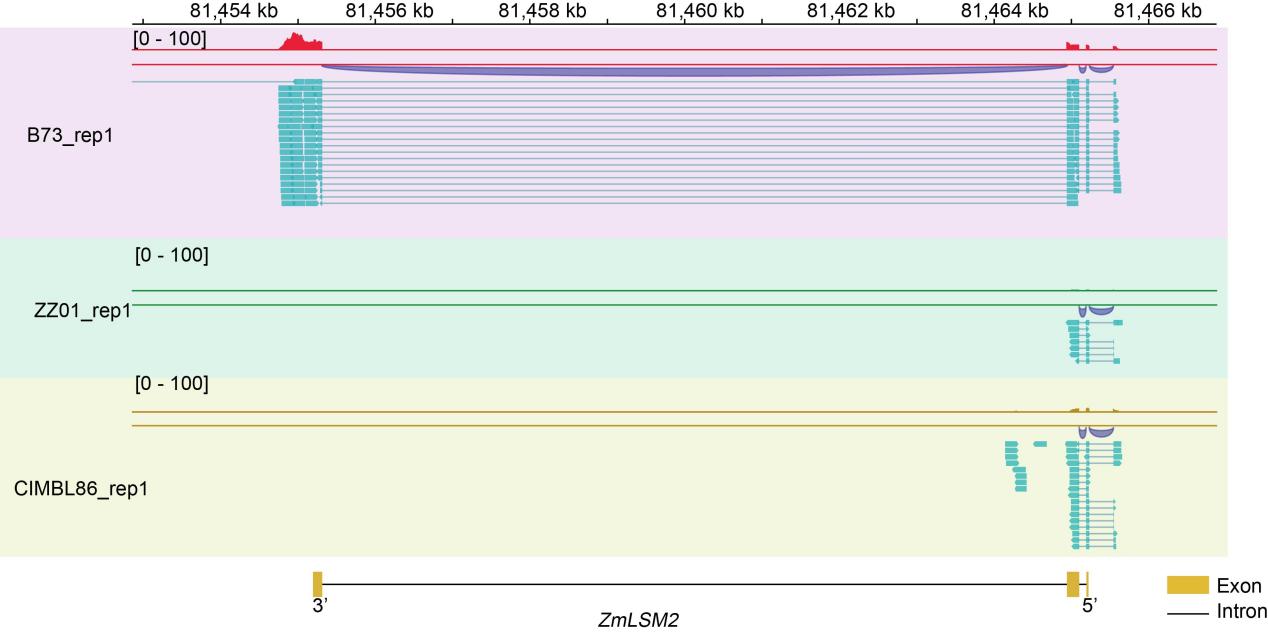
**

**Figure S17. Visualization of *ZmLSM2* transcriptional patterns and splicing events across different maize inbred lines.**

Genome browser view showing RNA-seq read coverage and splice junctions at the *ZmLSM2* locus for B73 (top), ZZ01 (middle), and CIMBL86 (bottom). The gene model is shown at the bottom, oriented from 5' (right) to 3' (left).
